# Supplementary material for: Sex-Specific Adaptations in Alzheimer’s Disease and Ischemic Stroke: A Longitudinal Study in Male and Female APPswe/PS1dE9 Mice
Source: Life (Basel). 2025 Feb 21;15(3):333. doi: 10.3390/life15030333 (PMC11944048; doi:10.3390/life15030333)
Supplement: Supplementary file 1 [file life-15-00333-s001.zip › life-3485392-supplementary.pdf]

Supplementary Materials

Sample size calculation

A power analysis was performed prior to the experiment, using an alpha level ($\alpha = 0.05$), statistical power ($1 - \beta = 0.80$), an assumption of equal-sized sample groups, and a calculated effect size of 1.79, resulting in a required sample size of n = 8 –15 per group (actual power: 0.93). The effect size was based on previous publications, using similar methodologies [1, 2]. To account for potential mortality, estimated rates for MCAO and sham procedures were considered, with 35% and 0% for wild-type mice and 60% and 10% for APP/PS1 mice, respectively. Based on these estimations, a total of 180 mice (90 males and 90 females, evenly distributed between wild-type and APP/PS1) were included. Mortality rates, based on previous studies [3], were primarily attributed to MCAO-related complications such as hemorrhage or, in APP/PS1 mice, prolonged seizures [4]. This sample size ensured that each experimental group retained 8 –15 animals.

1. Lohkamp, K.J., et al., *The impact of voluntary exercise on stroke recovery.* Frontiers in neuroscience, 2021. **15**: p. 695138.
2. Wiesmann, M., et al., *A specific dietary intervention to restore brain structure and function after ischemic stroke.* Theranostics, 2017. **7**(2): p. 493.
3. Kemppainen, S., et al., *Behavioral and neuropathological consequences of transient global ischemia in APP/PS1 Alzheimer model mice.* Behavioural brain research, 2014. **275**: p. 15-26.
4. Minkeviciene, R., et al., *Amyloid β-induced neuronal hyperexcitability triggers progressive epilepsy.* Journal of Neuroscience, 2009. **29**(11): p. 3453-3462

Supplementary Table S1. Number of animals per group at the start and end of the study, including mortality rate.

Group	initial animal count	final animal count	mortality (%)
**male WT sham**	17	14	18
**male WT stroke**	17	13	24
**male APP/PS1 sham**	17	9	47
**male APP/PS1 stroke**	19	8	58
**female WT sham**	17	15	12
**female WT stroke**	18	15	17
**female APP/PS1 sham**	15	10	33
**female APP/PS1 stroke**	22	9	59

Supplementary Table S2. Imaging sequences for the anatomical references, ASL, DTI, stroke RARE and Stoke DWI.

	Imaging method	Echo time (ms)	Repetition time	Image matrix	Field-of-view (mm)	Spatial resolution (µm/pixel)	No. of slices	Total acquisition time (min)
**Anatomical T2*w**	**GE**	7.357	865.086 ms	512 × 512	40 × 40	78 × 78 × 500	20 × 3	~8
**CBF**	**FAIR-ASL**	10.08	12 s	128 × 128	25 × 25	260 × 260 × 1000	1	~13
**Diffusion tensor imaging**	**6-shot spin-echo EPI**	21	7.75 s	128 × 128	20 × 20	156 × 156 × 500	20	~35
**Stroke RARE**	**RARE**	40	2.5 s	512 × 512	25.6 × 25.6	50 × 50 × 500	12	~4
**Stroke DWI**	**Spin-echo DW_Contrast**	27	2.25 s	128 × 128	12.8 × 12.8	100 × 100 × 500	9	~15

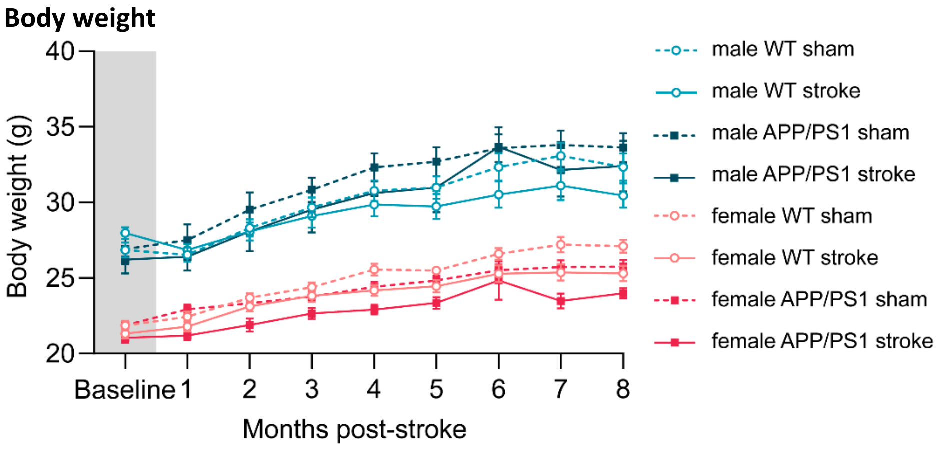

Supplementary Figure S1. Body weight changes from baseline over a period of 8 months, following either sham or stroke surgery in both male and female, wild-type (WT) and APP/PS1 mice. Data are presented as mean ± SEM.

Supplementary Table S3. Mean body weight ± SEM and group size (n) at baseline and over a period of 8 months, following either sham or stroke surgery, in both male and female, wild-type (WT), and APP/PS1 mice.

	male WT sham		male WT stroke		male APP/PS1 sham		male APP/PS1 stroke		female WT sham		female WT stroke		female APP/PS1 sham		female APP/PS1 stroke	
	mean ± SEM	n	mean ± SEM	n	mean ± SEM	n	mean ± SEM	n	mean ± SEM	n	mean ± SEM	n	mean ± SEM	n	mean ± SEM	n
**Baseline**	26.85 ± 0.49	14	26.91 ± 1	13	26.91 ± 1	7	26.79 ± 0.82	8	21.85 ± 0.34	15	21.32 ± 0.33	15	21.88 ± 0.22	10	21.04 ± 0.33	9
**Month 1**	26.54 ± 0.47	14	27.51 ± 1.05	13	27.51 ± 1.05	7	26.89 ± 0.89	8	22.45 ± 0.34	15	21.78 ± 0.34	15	22.94 ± 0.33	10	21.2 ± 0.32	9
**Month 2**	28.31 ± 0.58	14	29.51 ± 1.15	13	29.51 ± 1.15	7	29.08 ± 0.96	8	23.7 ± 0.29	15	23.12 ± 0.34	15	23.34 ± 0.33	10	21.9 ± 0.43	9
**Month 3**	29.68 ± 0.66	14	30.84 ± 0.81	13	30.84 ± 0.81	7	30.49 ± 1.31	8	24.39 ± 0.31	15	23.82 ± 0.4	15	23.78 ± 0.31	10	22.66 ± 0.36	9
**Month 4**	30.76 ± 0.68	14	32.31 ± 0.93	13	32.31 ± 0.93	7	31.75 ± 1.18	8	25.55 ± 0.38	15	24.18 ± 0.37	15	24.42 ± 0.33	10	22.92 ± 0.32	9
**Month 5**	30.99 ± 0.73	14	32.7 ± 0.95	13	32.7 ± 0.95	7	32.19 ± 1.25	8	25.51 ± 0.22	15	24.47 ± 0.41	15	24.85 ± 0.34	10	23.36 ± 0.38	9
**Month 6**	32.34 ± 0.91	14	33.6 ± 0.92	13	33.6 ± 0.92	7	33.54 ± 1.46	8	26.59 ± 0.39	15	25.29 ± 0.56	15	25.51 ± 0.39	10	24.84 ± 1.28	9
**Month 7**	33.09 ± 0.88	14	33.8 ± 0.96	13	33.8 ± 0.96	7	33.48 ± 1.33	8	27.21 ± 0.52	15	25.38 ± 0.54	15	25.73 ± 0.44	10	23.48 ± 0.49	9
**Month 8**	32.35 ± 0.91	14	33.64 ± 0.97	13	33.64 ± 0.97	7	33.63 ± 1.29	8	27.1 ± 0.44	15	25.31 ± 0.52	14	25.75 ± 0.45	10	23.99 ± 0.35	9

Supplementary Table S4. Results of body weight analysis using a linear mixed model, including all significant effects of fixed factors (sex, genotype, surgery, time) and their respective interactions. The table includes the direction of each effect, with numbers specifying the time point or time span at which each effect was observed. F-values, degrees of freedom, and p-values are provided.

**Effect**	**Direction**			**p-value**
**Genotype*Sex*Time**	1	WT	female<male	F(1,124)=59.19; p<0.001
	2		female<male	F(1,124)=64.97; p<0.001
	3		female<male	F(1,124)=79.16; p<0.001
	4		female<male	F(1,124)=84.11; p<0.001
	5		female<male	F(1,124)=81.91; p<0.001
	6		female<male	F(1,124)=85.41; p<0.001
	7		female<male	F(1,124)=95.59; p<0.001
	8		female<male	F(1,124)=75.64; p<0.001
	1	APP/PS1	female<male	F(1,124)=44.04; p<0.001
	2		female<male	F(0,124)=74.46; p<0.001
	3		female<male	F(0,124)=92.64; p<0.001
	4		female<male	F(0,124)=116.78; p<0.001
	5		female<male	F(0,124)=116.23; p<0.001
	6		female<male	F(0,124)=117.57; p<0.001
	7		female<male	F(0,124)=136.52; p<0.001
	8		female<male	F(0,124)=128.48; p<0.001
	4	male	APP/PS1>WT	F(1,124)=5.8; p<0.017
	5		APP/PS1>WT	F(1,124)=8.49; p<0.004
	6		APP/PS1>WT	F(1,124)=8.88; p<0.003
	7		APP/PS1>WT	F(1,124)=4.75; p<0.031
	8		APP/PS1>WT	F(1,124)=9.9; p<0.002
	7	female	APP/PS1<WT	F(0,124)=6.48; p<0.012
	8		APP/PS1<WT	F(0,124)=4.12; p<0.045
	1→2	male WT	increasing	F(7,594)=89; p<0.001
	1→8		increasing	F(7,594)=89; p<0.001
	2→3		increasing	F(7,594)=89; p<0.001
	3→4		increasing	F(7,594)=89; p<0.022
	5→6		increasing	F(7,594)=89; p<0.003

	1→2	male APP/PS1	increasing	F(7,594)=82.04; p<0.001
	1→8		increasing	F(7,594)=82.04; p<0.001
	2→3		increasing	F(7,594)=82.04; p<0.006
	3→4		increasing	F(7,594)=82.04; p<0.006
	1→2	female WT	increasing	F(7,594)=64.33; p<0.001
	1→8		increasing	F(7,594)=64.33; p<0.001
	5→6		increasing	F(7,594)=64.33; p<0.007
	1→8	female APP/PS1	increasing	F(7,594)=22.96; p<0.001
	5→6		increasing	F(7,594)=22.96; p<0.031
**Genotype*Surgery*Time**	6	WT	stroke<sham	F(1,124)=6.8; p<0.01
	7		stroke<sham	F(1,124)=10.25; p<0.002
	8		stroke<sham	F(1,124)=9.4; p<0.003
	1→2	WT sham	increasing	F(7,594)=110.35; p<0.001
	1→8		increasing	F(7,594)=110.35; p<0.001
	2→3		increasing	F(7,594)=110.35; p<0.003
	3→4		increasing	F(7,594)=110.35; p<0.001
	5→6		increasing	F(7,594)=110.35; p<0.001
	1→2	WT stroke	increasing	F(7,594)=49.39; p<0.001
	1→8		increasing	F(7,594)=49.39; p<0.001
	2→3		increasing	F(7,594)=49.39; p<0.038
	1→2	APP/PS1 sham	increasing	F(7,594)=46.95; p<0.017
	1→8		increasing	F(7,594)=46.95; p<0.001
	1→2	WT stroke	increasing	F(7,594)=52.34; p<0.001
	1→8		increasing	F(7,594)=52.34; p<0.001
	2→3		increasing	F(7,594)=52.34; p<0.047
	5→6		increasing	F(7,594)=52.34; p<0.001

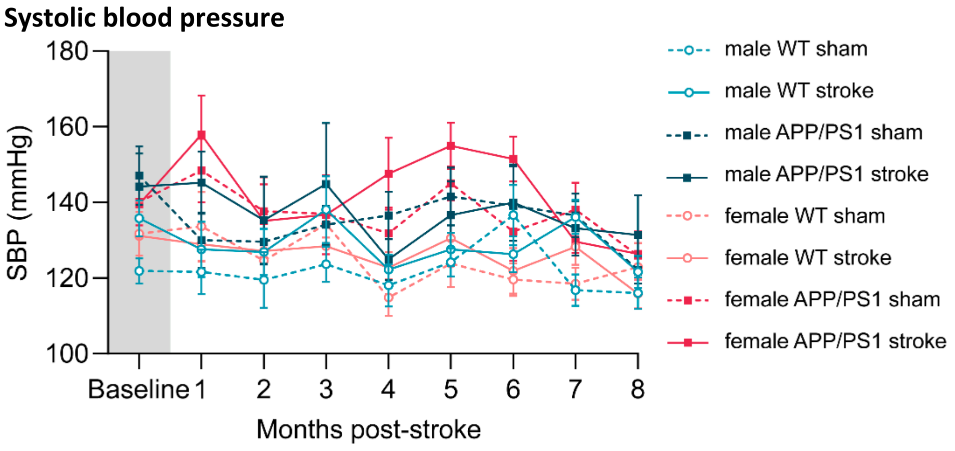

Supplementary Figure S2. Systolic blood pressure changes from baseline over a period of 8 months, following either sham or stroke surgery in both male and female, wild-type (WT) and APP/PS1 mice. Data are presented as mean ± SEM.

Supplementary Table S5. Mean systolic blood pressure ± SEM and group size (n) at baseline and over a period of 8 months, following either sham or stroke surgery, in both male and female, wild-type (WT), and APP/PS1 mice.

	male WT sham		male WT stroke		male APP/PS1 sham		male APP/PS1 stroke		female WT sham		female WT stroke		female APP/PS1 sham		female APP/PS1 stroke	
	mean ± SEM	n	mean ± SEM	n	mean ± SEM	n	mean ± SEM	n	mean ± SEM	n	mean ± SEM	n	mean ± SEM	n	mean ± SEM	n
**Baseline**	121.87 ± 0.5	13	135.84 ± 0.39	13	147.06 ± 0.98	7	144.2 ± 0.63	8	131.75 ± 0.41	12	131.13 ± 0.43	11	140.14 ± 0.19	10	139.52 ± 0.32	9
**Month 1**	121.68 ± 0.51	13	127.54 ± 0.4	12	130.05 ± 1.18	5	145.19 ± 0.82	8	133.69 ± 0.36	13	128.91 ± 0.36	13	148.45 ± 0.23	9	157.94 ± 0.36	8
**Month 2**	119.53 ± 0.67	11	126.88 ± 0.47	10	129.6 ± 1.13	5	135.28 ± 1	5	124.59 ± 0.39	10	127.17 ± 0.42	11	137.63 ± 0.2	9	135.1 ± 0.33	8
**Month 3**	123.7 ± 0.35	10	138.12 ± 0.47	12	134.08 ± 1.24	6	144.84 ± 1.05	5	134.28 ± 0.4	11	128.41 ± 0.41	12	136.99 ± 0.23	10	136.71 ± 0.67	5
**Month 4**	118.07 ± 0.45	12	122.21 ± 0.7	10	136.61 ± 1.05	7	124.96 ± 1.17	5	114.88 ± 0.36	12	122.74 ± 0.36	13	131.8 ± 0.33	8	147.54 ± 0.44	6
**Month 5**	124.17 ± 0.46	12	127.57 ± 0.86	12	141.56 ± 1.21	6	136.67 ± 0.98	7	123.83 ± 0.36	12	130.53 ± 0.37	14	145 ± 0.32	9	154.98 ± 0.33	9
**Month 6**	136.66 ± 0.46	12	126.35 ± 1.06	12	139.07 ± 1.06	7	139.93 ± 0.78	8	119.66 ± 0.31	12	121.87 ± 0.31	13	132.32 ± 0.36	9	151.51 ± 0.3	8
**Month 7**	116.79 ± 0.44	11	136.11 ± 1.06	12	136.61 ± 1.09	7	133.18 ± 1.06	6	118.49 ± 0.34	13	128.3 ± 0.36	12	138.1 ± 0.31	10	129.62 ± 0.28	5
**Month 8**	116.03 ± 0.4	13	121.63 ± 0.78	12	122.13 ± 1.38	5	131.39 ± 0.96	6	122.96 ± 0.38	9	115.92 ± 0.42	12	125.96 ± 0.39	7	126.41 ± 0.26	7

Supplementary Table S6. Results of systolic blood pressure analysis using a linear mixed model, including all significant effects of fixed factors (sex, genotype, surgery, time) and their respective interactions. The table includes the direction of each effect, with numbers specifying the time point or time span at which each effect was observed. F-values, degrees of freedom, and p-values are provided.

**Effect**	**Direction**		**p-value**
Genotype	baseline	APP/PS1>WT	$F(1,75)=7.05$; $p<0.01$
	1-8	APP/PS1>WT	$F(1,85)=38.81$; $p<0.001$

Digital ventilated cages – Distance

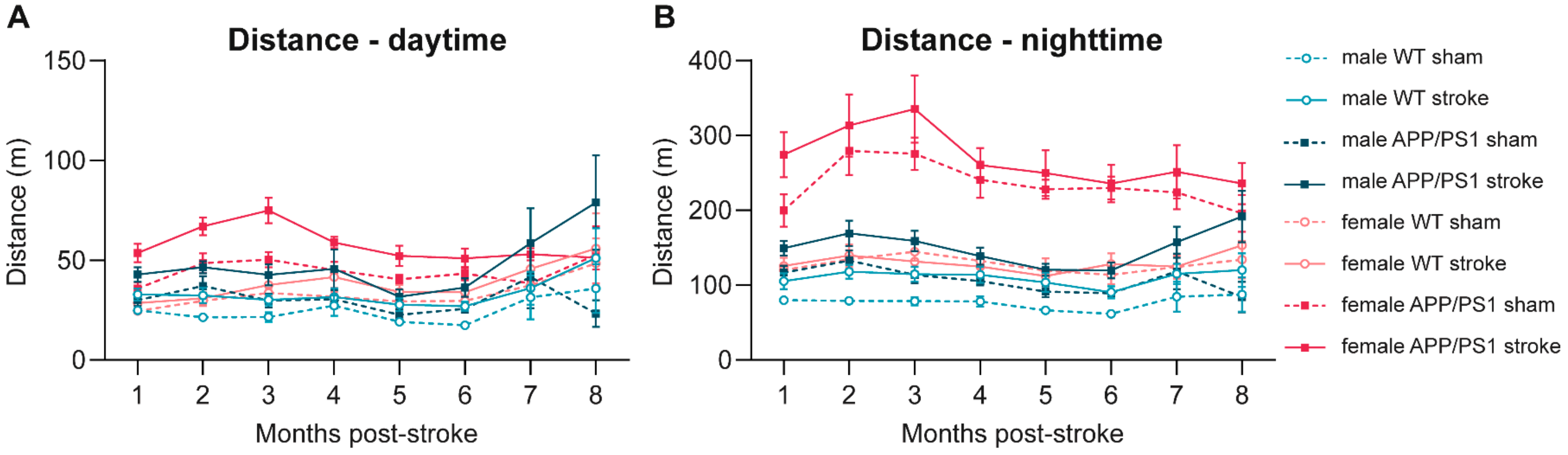

Supplementary Figure S3. Walking distance monitored in digital ventilated cages over a period of 8 months, following either sham or stroke surgery in both male and female, wild-type (WT) and APP/PS1 mice. Data are presented as mean ± SEM.

Supplementary Table S7. Mean walking distance ± SEM and group size (n) at baseline and over a period of 8 months, following either sham or stroke surgery, in both male and female, wild-type (WT), and APP/PS1 mice.

		male WT sham		male WT stroke		male APP/PS1 sham		male APP/PS1 stroke		female WT sham		female WT stroke		female APP/PS1 sham		female APP/PS1 stroke	
		mean ± SEM	n	mean ± SEM	n	mean ± SEM	n	mean ± SEM	n	mean ± SEM	n	mean ± SEM	n	mean ± SEM	n	mean ± SEM	n
**Month 1**	**Distance - daytime**	24.86 ± 1.73	11	32.77 ± 3.09	13	29.88 ± 2.93	6	42.79 ± 3.72	8	24.53 ± 1.86	14	28.55 ± 1.87	13	35.9 ± 3.2	9	53.63 ± 4.55	9
**Month 2**	**Distance - daytime**	21.42 ± 1.2	11	32.38 ± 3.81	13	37.13 ± 5	6	46.44 ± 3.12	8	29.59 ± 2.55	14	30.96 ± 3.08	13	48.6 ± 5.01	9	66.93 ± 4.44	9
**Month 3**	**Distance - daytime**	21.53 ± 2.35	11	30.18 ± 2.74	13	29.74 ± 3.52	6	42.73 ± 5.27	8	33.68 ± 2.65	14	37.57 ± 3.91	13	50.27 ± 3.82	9	74.87 ± 6.4	9
**Month 4**	**Distance - daytime**	27.19 ± 5.22	11	31.54 ± 4.39	13	30.48 ± 4.6	6	45.7 ± 9.64	8	31.72 ± 2.51	14	41.51 ± 5.41	13	45.04 ± 4.15	9	58.91 ± 2.83	9
**Month 5**	**Distance - daytime**	19.13 ± 1.31	11	27.72 ± 3.19	13	22.65 ± 2.75	6	31.71 ± 3.65	8	29.33 ± 3.17	14	34.05 ± 3.52	13	40.37 ± 2.35	9	52.22 ± 5.05	9
**Month 6**	**Distance - daytime**	17.47 ± 1.35	11	26.87 ± 2.37	13	25.67 ± 1.93	6	36.32 ± 4.56	8	29.65 ± 2.73	14	33.97 ± 3.11	13	43.32 ± 3.36	9	50.86 ± 4.94	9
**Month 7**	**Distance - daytime**	31.43 ± 10.93	11	36.06 ± 8.39	12	41.51 ± 15.66	5	58.58 ± 17.43	7	37.37 ± 7.38	13	45.61 ± 10.99	11	38.38 ± 3.79	8	52.96 ± 3.01	8
**Month 8**	**Distance - daytime**	35.77 ± 12.26	11	51.15 ± 15.04	12	23.26 ± 6.69	4	78.87 ± 23.63	6	48.45 ± 12.62	13	55.92 ± 17.77	9	52.74 ± 14.44	8	51.31 ± 5.91	5
**Month 1**	**Distance - nighttime**	79.91 ± 3.76	11	105.26 ± 10.79	13	116.75 ± 9.24	6	149.22 ± 9.89	8	120.13 ± 7.1	14	125.62 ± 11	13	199.75 ± 21.56	9	274.48 ± 30.39	9
**Month 2**	**Distance - nighttime**	79.19 ± 5.37	11	117.85 ± 9.33	13	132.69 ± 13.71	6	169.44 ± 17.15	8	134.33 ± 12.63	14	139.65 ± 14.74	13	279.26 ± 32.51	9	313.19 ± 41.74	9
**Month 3**	**Distance - nighttime**	78.63 ± 5.57	11	114.45 ± 9.85	13	113.8 ± 11.13	6	158.99 ± 13.67	8	144.59 ± 11.99	14	131.49 ± 13.38	13	275.77 ± 21.54	9	335.47 ± 45.15	9
**Month 4**	**Distance - nighttime**	78.18 ± 6.71	11	113.6 ± 10.97	13	105.51 ± 5.72	6	138.87 ± 11.75	8	132.21 ± 10.43	14	124.67 ± 13.7	13	241.03 ± 24.34	9	260.21 ± 23.18	9
**Month 5**	**Distance - nighttime**	66.29 ± 3.3	11	103.46 ± 12.75	13	91.46 ± 7.44	6	120.44 ± 8.24	8	119.28 ± 16.71	14	112.1 ± 11.43	13	228.07 ± 12.99	9	249.64 ± 30.79	9
**Month 6**	**Distance - nighttime**	61.57 ± 3.08	11	90.33 ± 8.15	13	89.27 ± 3.96	6	119.52 ± 10.88	8	113.75 ± 12.51	14	128.29 ± 14.57	13	229.7 ± 15.51	9	235.75 ± 24.94	9
**Month 7**	**Distance - nighttime**	84.44 ± 19.97	11	114.96 ± 13.83	12	118.53 ± 23.83	5	157.44 ± 20.79	7	124.32 ± 14.93	13	125 ± 16.46	11	224.1 ± 22.77	8	251.49 ± 35.55	8
**Month 8**	**Distance - nighttime**	87.36 ± 22.91	11	120.18 ± 22.53	12	84.3 ± 20.4	4	191.9 ± 34.5	6	134.08 ± 24.83	13	153.24 ± 35.46	9	195.85 ± 24.8	8	235.79 ± 27.5	5

Supplementary Table S8. Results of walking distance analysis using a linear mixed model, including all significant effects of fixed factors (sex, genotype, surgery, time) and their respective interactions. The table includes the direction of each effect, with numbers specifying the time point or time span at which each effect was observed. F-values, degrees of freedom, and p-values are provided.

Time	Effect	Direction			p-value
**daytime**	**Sex**	female>male			F(1,80)=10.66; p<0.002
	**Genotype**	APP/PS1>WT			F(1,80)=23.88; p<0.001
	**Surgery**	stroke>sham			F(1,80)=14.36; p<0.001
**nighttime**	**Surgery**	stroke>sham			F(1,78)=6.41; p<0.013
	**Sex*Genotype*Time**	2	WT	female>male	F(1,221)=5.71; p<0.018
		3		female>male	F(1,221)=6.8; p<0.01
		4		female>male	F(1,221)=4.17; p<0.042

		6		female>male	F(1,221)=7.94; p<0.005
		8		female>male	F(1,221)=5.7; p<0.018
		1	APP/PS1	female>male	F(1,221)=25.99; p<0.001
		2		female>male	F(1,221)=50.46; p<0.001
		3		female>male	F(1,221)=68.2; p<0.001
		4		female>male	F(1,221)=39.58; p<0.001
		5		female>male	F(1,221)=42.61; p<0.001
		6		female>male	F(1,221)=39.66; p<0.001
		7		female>male	F(1,221)=19.37; p<0.001
		8		female>male	F(1,221)=5.73; p<0.017
		1	male	APP/PS1>WT	F(1,222)=4.57; p<0.034
		2		APP/PS1>WT	F(1,222)=7.61; p<0.006
		3		APP/PS1>WT	F(1,222)=4.55; p<0.034
		7		APP/PS1>WT	F(1,222)=4.13; p<0.043
		8		APP/PS1>WT	F(1,222)=3.98; p<0.047
		1	female	APP/PS1>WT	F(1,222)=43.19; p<0.001
		2		APP/PS1>WT	F(1,222)=84.05; p<0.001
		3		APP/PS1>WT	F(1,222)=92.73; p<0.001
		4		APP/PS1>WT	F(1,222)=49.22; p<0.001
		5		APP/PS1>WT	F(1,222)=50.03; p<0.001
		6		APP/PS1>WT	F(1,222)=41.41; p<0.001
		7		APP/PS1>WT	F(1,222)=35.71; p<0.001
		8		APP/PS1>WT	F(1,222)=8.58; p<0.004
		1→2	female APP/PS1	increasing	F(7,530)=22.95; p<0.001
		5→6		increasing	F(7,530)=22.95; p<0.001

Morris water maze - Acquisition

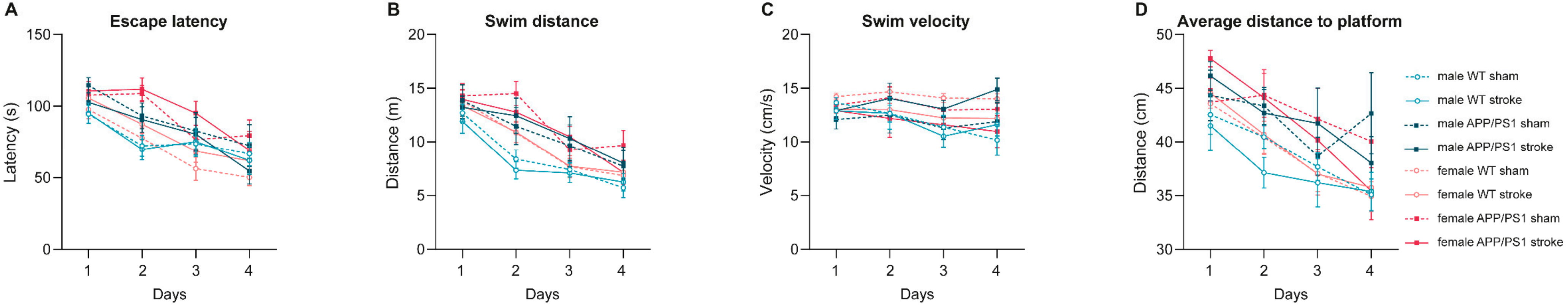

Supplementary Figure S4. Spatial learning performance during the 4-day acquisition phase of the Morris water maze in male and female, wild-type (WT), and APP/PS1 mice, assessed 8 months post-surgery at 12 months of age. Performance metrics include: (A) latency to find the hidden platform, (B) total swim distance, (C) swim velocity, and (D) average distance to the platform. Data are presented as mean ± SEM.

Supplementary Table S9. Raw data from the 4-day acquisition phase of the Morris water maze, displaying mean ± SEM and group size (n) for latency to locate the hidden platform, total swim distance, swim velocity, and average distance to the platform in male and female wild-type (WT) and APP/PS1 mice, assessed 8 months post-surgery at 12 months of age.

Acquisition		male WT sham		male WT stroke		male APP/PS1 sham		male APP/PS1 stroke		female WT sham		female WT stroke		female APP/PS1 sham		female APP/PS1 stroke	
		**mean ± SEM**	**n**	**mean ± SEM**	**n**	**mean ± SEM**	**n**	**mean ± SEM**	**n**	**mean ± SEM**	**n**	**mean ± SEM**	**n**	**mean ± SEM**	**n**	**mean ± SEM**	**n**
**Day 1**	**Latency (s)**	94.43 ± 6.29	14	95.08 ± 7.16	12	114.49 ± 5.32	5	102.68 ± 6.92	7	97.56 ± 6.42	15	105.63 ± 4.2	14	107.58 ± 6.82	8	110.61 ± 6.64	5
**Day 2**	**Latency (s)**	72.16 ± 7.37	14	69.71 ± 7.28	12	93.2 ± 9.09	5	90.68 ± 9.01	7	77.48 ± 7.46	15	87.36 ± 6.36	14	108.85 ± 5.69	8	111.79 ± 7.87	5
**Day 3**	**Latency (s)**	73.64 ± 9	14	75.1 ± 9.45	12	82.62 ± 12.82	5	80.4 ± 11.93	7	56.24 ± 8.11	15	68.38 ± 7.74	14	76.58 ± 10.14	8	95.01 ± 8.47	5
**Day 4**	**Latency (s)**	66.62 ± 8.13	14	61.96 ± 9.75	12	72.38 ± 14.79	5	54.5 ± 9.04	7	50.16 ± 5.75	15	61.71 ± 8.2	14	79.4 ± 11.02	8	69.07 ± 13.23	5
**Day 1**	**Distance (m)**	12.68 ± 0.84	14	11.9 ± 1.11	12	13.84 ± 1.45	5	13.22 ± 1.06	7	13.79 ± 1.01	15	13.38 ± 0.63	14	14.28 ± 1.14	8	13.97 ± 0.94	5
**Day 2**	**Distance (m)**	8.37 ± 0.85	14	7.34 ± 0.79	12	11.45 ± 1.73	5	12.44 ± 1.63	7	10.85 ± 0.96	15	10.91 ± 1	14	14.5 ± 1.13	8	12.77 ± 1.8	5
**Day 3**	**Distance (m)**	7.4 ± 0.69	14	7.08 ± 0.9	12	9.64 ± 1.96	5	10.3 ± 2.03	7	7.66 ± 1.03	15	7.72 ± 0.73	14	9.23 ± 1.08	8	10.47 ± 0.96	5
**Day 4**	**Distance (m)**	5.71 ± 0.92	14	6.23 ± 0.81	12	7.71 ± 1.46	5	8.01 ± 1.49	7	6.85 ± 0.8	15	7.15 ± 0.97	14	9.67 ± 1.42	8	7.15 ± 1.12	5
**Day 1**	**Velocity (cm/s)**	13.66 ± 0.46	14	12.89 ± 0.8	12	12.09 ± 0.88	5	12.91 ± 0.57	7	14.22 ± 0.33	15	13.03 ± 0.4	14	13.42 ± 0.47	8	12.91 ± 0.87	5
**Day 2**	**Velocity (cm/s)**	12.65 ± 0.78	14	12.67 ± 1.48	12	12.49 ± 1.67	5	14.05 ± 1.42	7	14.67 ± 0.39	15	13.01 ± 0.58	14	14.12 ± 1.03	8	12.18 ± 1.78	5
**Day 3**	**Velocity (cm/s)**	11.37 ± 1.11	14	10.53 ± 1.05	12	11.3 ± 1.1	5	13.07 ± 0.8	7	14.08 ± 0.42	15	12.24 ± 0.71	14	12.98 ± 1.26	8	11.59 ± 1.33	5
**Day 4**	**Velocity (cm/s)**	10.18 ± 1.44	14	11.61 ± 1.07	12	11.87 ± 1.77	5	14.88 ± 1.08	7	14.02 ± 0.47	15	12.19 ± 0.7	14	13.02 ± 0.77	8	10.96 ± 1.52	5
**Day 1**	**Distance to platform (mean in cm)**	42.53 ± 1.84	14	41.51 ± 2.31	12	44.34 ± 2.35	5	46.14 ± 1.32	7	43.56 ± 1.16	15	44.54 ± 1.42	14	43.71 ± 1.26	8	47.76 ± 0.77	5
**Day 2**	**Distance to platform (mean in cm)**	40.46 ± 1.07	14	37.12 ± 1.43	12	43.35 ± 1.74	5	42.7 ± 2.19	7	40.43 ± 1.52	15	40.7 ± 1.89	14	44.36 ± 2.06	8	44.12 ± 2.62	5
**Day 3**	**Distance to platform (mean in cm)**	37.66 ± 1.27	14	36.18 ± 2.25	12	38.66 ± 1.79	5	41.72 ± 3.3	7	37.05 ± 2.02	15	36.99 ± 1.6	14	42.14 ± 2.89	8	40.16 ± 1.75	5
**Day 4**	**Distance to platform (mean in cm)**	35.08 ± 1.47	14	35.35 ± 1.78	12	42.65 ± 3.79	5	38.02 ± 2.47	7	34.93 ± 1.37	15	35.74 ± 2.23	14	40.05 ± 2.46	8	35.42 ± 2.69	5
**Day 1**	**Distance to platform (total in m)**	1073.24 ± 87.89	14	1049.05 ± 103.76	12	1293.34 ± 98.05	5	1236.35 ± 96.53	7	1105.49 ± 83.14	15	1216.23 ± 69.56	14	1183.82 ± 91.31	8	1319.9 ± 78.84	5
**Day 2**	**Distance to platform (total in m)**	748.72 ± 84.6	14	702.28 ± 104.93	12	1088.76 ± 128.61	5	1058.42 ± 139.53	7	846.23 ± 94.99	15	938.88 ± 88.52	14	1250.11 ± 87.34	8	1234.44 ± 148.12	5
**Day 3**	**Distance to platform (total in m)**	741.73 ± 100.49	14	758.36 ± 118.52	12	902.53 ± 143.23	5	922.79 ± 172.79	7	586.43 ± 107.02	15	696.63 ± 93.54	14	889.92 ± 156.77	8	1007.28 ± 122.55	5
**Day 4**	**Distance to platform (total in m)**	621.94 ± 92	14	611.35 ± 109.5	12	839.85 ± 219.76	5	611.34 ± 122.71	7	488.64 ± 60.18	15	611.09 ± 107.21	14	874.45 ± 145.73	8	729.74 ± 151.54	5

Supplementary Table S10. Results of the linear mixed model analysis for the 4-day acquisition phase data (latency to locate the hidden platform, total swim distance, swim velocity, and average distance to the platform), highlighting all significant effects of fixed factors (sex, genotype, surgery, time), and their respective interactions. The table specifies the direction of each effect, with numbers specifying the time point or time span where the effect was observed. F-values, degrees of freedom, and p-values are included for each effect.

	**Latency**			**Swim distance**			**Swim velocity**				**Average distance to platform**		
	**Direction**		**p-value**	**Direction**		**p-value**	**Direction**			**p-value**	**Direction**		**p-value**
**Sex**				female>male		F(1,76)=7.16; p<0.009							
**Genotype**	APP/PS1>WT		F(1,78)=10.99; p<0.001	APP/PS1>WT		F(1,77)=19.04; p<0.001					APP/PS1>WT		F(1,78)=11.08; p<0.001
**Time**	1→2	decreasing	F(3,237)=43.72; p<0.001	1→2	decreasing	F(3,237)=63.37; p<0.001					1→2	decreasing	F(3,237)=34.47; p<0.002
	1→4	decreasing	F(3,237)=43.72; p<0.001	1→4	decreasing	F(3,237)=63.37; p<0.001					1→4	decreasing	F(3,237)=34.47; p<0.001
	2→3	decreasing	F(3,237)=43.72; p<0.005	2→3	decreasing	F(3,237)=63.37; p<0.001					2→3	decreasing	F(3,237)=34.47; p<0.003
	3→4	decreasing	F(3,237)=43.72; p<0.04										
**Sex*Genotype *Time**							3	WT	female>male	F(1,140)=7.54; p<0.007			
							4		female>male	F(1,140)=8.15; p<0.005			
							4	male	APP/PS1>WT	F(1,140)=7.21; p<0.008			
							1→4	male WT	decreasing	F(3,228)=11.77; p<0.001			
							2→3		decreasing	F(3,228)=11.77; p<0.006			

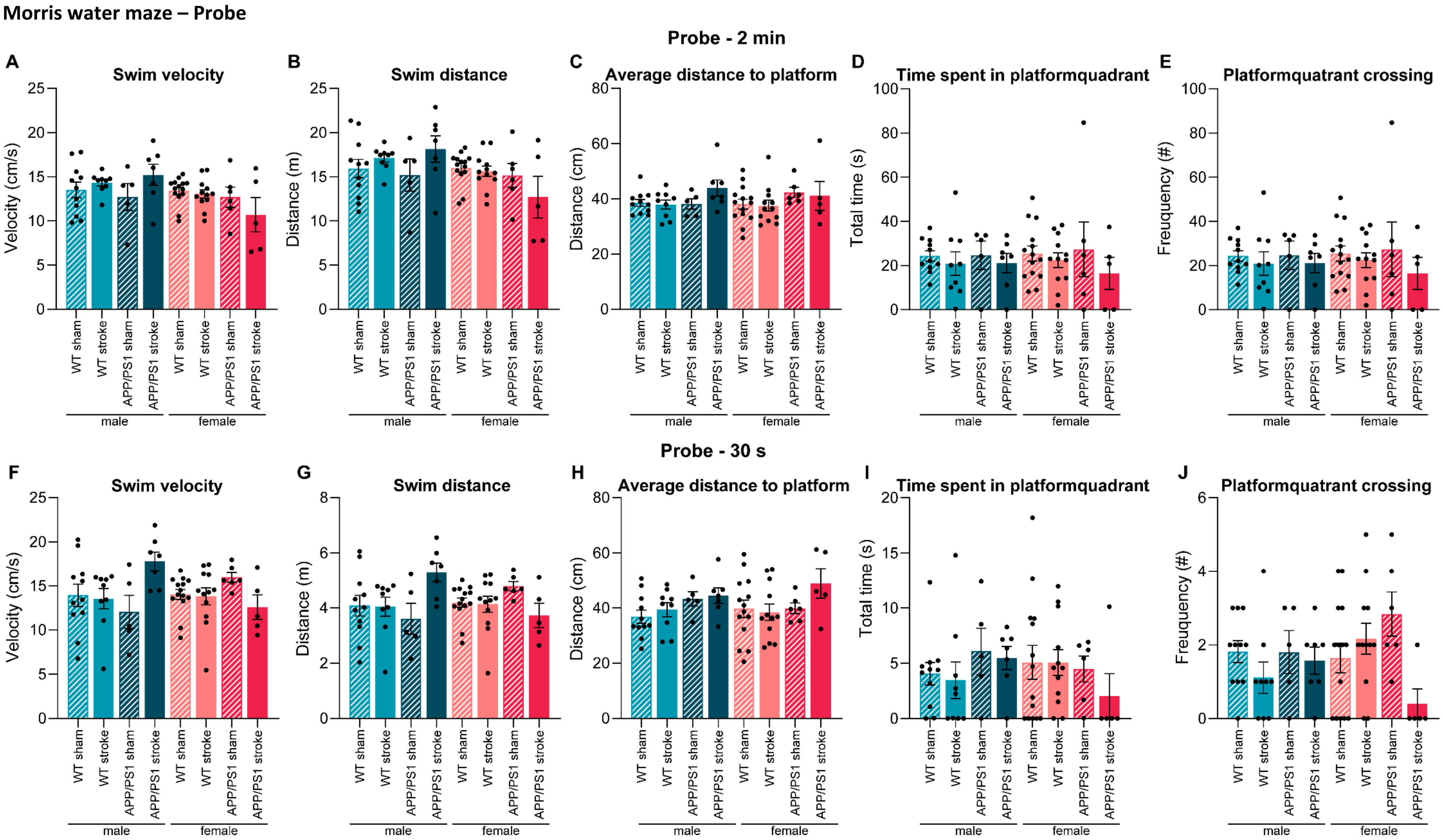

Supplementary Figure S5. Spatial memory assessment during the probe trial of the Morris water maze in both male and female, wild-type (WT), and APP/PS1 mice, conducted 8 months post-surgery at 12 months of age. Performance metrics include: (A) swim velocity, (B) swim distance, (C) average distance to the platform, (D) time spent in the platform quadrant, and (E) platform quadrant crossings over the full 2-minute trial. Additionally, an analysis for the initial 30 seconds of the probe trial was performed: (F) swim velocity, (G) swim distance, (H) average distance to the platform, (I) time spent in the platform quadrant, and (J) platform quadrant crossings. Data are presented as mean ± SEM.

Supplementary Table S11. Raw data from the probe trial of the Morris water maze, showing mean ± SEM and group size (n) for swim velocity, swim distance, average distance to the platform, time spent in the platform quadrant, and platform quadrant crossings in male and female wild-type (WT) and APP/PS1 mice, assessed 8 months post-surgery at 12 months of age. Data for both the full 2-minute probe trial and the initial 30 s are presented.

Probe		male WT sham		male WT stroke		male APP/PS1 sham		male APP/PS1 stroke		female WT sham		female WT stroke		female APP/PS1 sham		female APP/PS1 stroke	
		**mean ± SEM**	**n**	**mean ± SEM**	**n**	**mean ± SEM**	**n**	**mean ± SEM**	**n**	**mean ± SEM**	**n**	**mean ± SEM**	**n**	**mean ± SEM**	**n**	**mean ± SEM**	**n**
**2 min**	**Swim velocity (cm/s)**	13.52 ± 0.86	11	14.31 ± 0.38	9	12.7 ± 1.51	5	15.21 ± 1.19	7	13.41 ± 0.4	14	13.05 ± 0.49	12	12.7 ± 1.13	6	10.69 ± 1.94	5
	**Swim distance (m)**	15.93 ± 1.04	11	17.12 ± 0.45	9	15.19 ± 1.81	5	18.13 ± 1.5	7	16.01 ± 0.49	14	15.6 ± 0.59	12	15.15 ± 1.34	6	12.7 ± 2.37	5
	**Average distance to platform (cm)**	38.55 ± 1.25	11	37.97 ± 1.65	9	38.1 ± 1.92	5	43.84 ± 2.98	7	38.1 ± 1.79	14	37.49 ± 1.98	12	42.31 ± 1.81	6	41.08 ± 5.24	5
	**Time spent in platformquadrant (s)**	24.41 ± 2.31	11	20.89 ± 5.3	9	24.67 ± 6.41	5	21.16 ± 4.39	7	25.39 ± 3.42	14	22.44 ± 3.33	12	27.35 ± 12.36	6	16.42 ± 7.27	5
	**Platformquadrant crossing (frequency)**	6.73 ± 0.38	11	5.78 ± 1.12	9	6.6 ± 1.78	5	5.71 ± 1.38	7	6.29 ± 0.76	14	7.08 ± 0.72	12	6.5 ± 1.75	6	3.8 ± 1.96	5
**30 s**	**Swim velocity (cm/s)**	13.91 ± 1.26	11	13.53 ± 1.14	9	12.05 ± 1.87	5	17.79 ± 1.06	7	14.02 ± 0.56	14	13.8 ± 0.97	12	15.96 ± 0.55	6	12.58 ± 1.4	5
	**Swim distance (m)**	4.08 ± 0.37	11	4.04 ± 0.34	9	3.61 ± 0.56	5	5.29 ± 0.34	7	4.19 ± 0.17	14	4.13 ± 0.29	12	4.78 ± 0.16	6	3.73 ± 0.44	5
	**Average distance to platform (cm)**	36.84 ± 2.37	11	39.38 ± 2.54	9	43.23 ± 2.63	5	44.42 ± 2.8	7	39.65 ± 3.13	14	38.41 ± 2.93	12	39.81 ± 1.93	6	48.89 ± 5.39	5
	**Time spent in platformquadrant (s)**	4.06 ± 1.02	11	3.46 ± 1.64	9	6.08 ± 2.1	5	5.46 ± 1.06	7	5.07 ± 1.53	14	5.06 ± 1.17	12	4.48 ± 1.18	6	2.02 ± 2.02	5
	**Platformquadrant crossing (frequency)**	1.82 ± 0.3	11	1.11 ± 0.42	9	1.8 ± 0.58	5	1.57 ± 0.37	7	1.64 ± 0.4	14	2.17 ± 0.42	12	2.83 ± 0.6	6	0.4 ± 0.4	5

Supplementary Table S12. Results of the linear mixed model analysis for the full 2-minute probe trial data, including swim velocity, swim distance, average distance to the platform, time spent in the platform quadrant, and platform quadrant crossings. The table highlights all significant effects of fixed factors (sex, genotype, surgery) and their respective interactions. F-values, degrees of freedom, and p-values are included for each effect.

	Swim velocity	
	**Direction**	**p-value**
**Sex**	female<male	F(1,65)=4.07; p<0.048

Supplementary Table S13. Results of the linear mixed model analysis for the full 2-minute probe trial data, including swim velocity, swim distance, average distance to the platform, time spent in the platform quadrant, and platform quadrant crossings. The table highlights all significant effects of fixed factors (sex, genotype, surgery) and their respective interactions. F-values, degrees of freedom, and p-values are included for each effect.

	Swim velocity			Swim distance			Average distance to platform	
	**Direction**		**p-value**	**Direction**		**p-value**	**Direction**	**p-value**
**Genotype**							APP/PS1>WT	F(1,67)=5.36; p<0.024
**Sex*Surgery**	stroke	female<male	F(1,61)=4.52; p<0.038	stroke	female<male	F(1,61)=4.43; p<0.04		
	male	stroke>sham	F(1,61)=5.23; p<0.026	male	stroke>sham	F(1,61)=5.43; p<0.023		

Morris water maze – Cognitive score

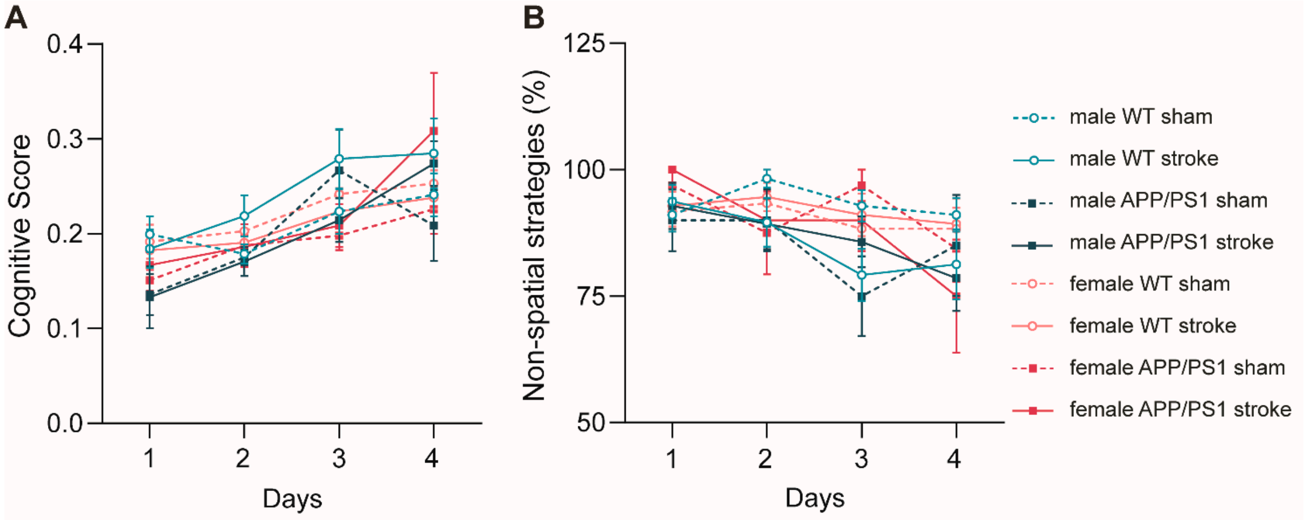

Supplementary Figure S6. Cognitive score (A) and percentage of non-spatial search strategies (B) during the 4-day acquisition phase of the Morris water maze in male and female, wild-type (WT), and APP/PS1 mice. Data are presented as mean ± SEM.

Supplementary Table S14. Raw data from the acquisition trial of the Morris water maze, showing mean ± SEM and group size (n) for cognitive scores and percentage of non-spatial search strategies in male and female wild-type (WT) and APP/PS1 mice, assessed 8 months post-surgery at 12 months of age. Data for both the full 2-minute probe trial and the initial 30 s are presented.

Acquisition		male WT sham		male WT stroke		male APP/PS1 sham		male APP/PS1 stroke		female WT sham		female WT stroke		female APP/PS1 sham		female APP/PS1 stroke	
		mean ± SEM	n	mean ± SEM	n	mean ± SEM	n	mean ± SEM	n	mean ± SEM	n	mean ± SEM	n	mean ± SEM	n	mean ± SEM	n
Day 1	Cognitive Score	0.2 ± 0.02	14	0.14 ± 0.02	12	0.18 ± 0.02	5	0.13 ± 0.03	7	0.19 ± 0.02	15	0.18 ± 0.02	14	0.15 ± 0.01	8	0.17 ± 0	5
Day 2	Cognitive Score	0.18 ± 0.01	14	0.18 ± 0.02	12	0.22 ± 0.01	5	0.17 ± 0.02	7	0.2 ± 0.01	15	0.19 ± 0.01	14	0.19 ± 0.02	8	0.19 ± 0.01	5
Day 3	Cognitive Score	0.22 ± 0.02	14	0.27 ± 0.03	12	0.28 ± 0.04	5	0.21 ± 0.02	7	0.24 ± 0.03	15	0.22 ± 0.02	14	0.2 ± 0.02	8	0.21 ± 0.02	5
Day 4	Cognitive Score	0.24 ± 0.02	14	0.21 ± 0.04	12	0.28 ± 0.04	5	0.27 ± 0.02	7	0.25 ± 0.03	15	0.24 ± 0.03	14	0.23 ± 0.03	8	0.31 ± 0.06	5
Day 1	Non-spatial search strategies (%)	91.07 ± 3.32	14	90 ± 3.26	12	93.75 ± 6.12	5	92.86 ± 4.61	7	91.67 ± 3.98	15	92.86 ± 4.08	14	96.88 ± 3.13	8	100 ± 0	5
Day 2	Non-spatial search strategies (%)	98.21 ± 1.79	14	90 ± 4.82	12	89.58 ± 6.12	5	89.29 ± 5.05	7	93.33 ± 2.95	15	94.64 ± 2.85	14	87.5 ± 8.18	8	90 ± 6.12	5
Day 3	Non-spatial search strategies (%)	92.86 ± 3.13	14	75 ± 5.18	12	79.17 ± 7.91	5	85.71 ± 5.05	7	88.33 ± 4.13	15	91.07 ± 4.23	14	96.88 ± 3.13	8	90 ± 6.12	5
Day 4	Non-spatial search strategies (%)	91.07 ± 3.32	14	85 ± 6.97	12	81.25 ± 10	5	78.57 ± 6.52	7	88.33 ± 4.13	15	89.29 ± 5.05	14	84.38 ± 6.58	8	75 ± 11.18	5

Supplementary Table S15. Results of the linear mixed model analysis for the cognitive score during the 4-day acquisition phase data, highlighting all significant effects of fixed factors (sex, genotype, surgery, time), and their respective interactions. The table specifies the direction of each effect, with numbers specifying the time point or time span where the effect was observed. F-values, degrees of freedom, and p-values are included for each effect.

	Cognitive score	
	**Direction**	**p-value**
**Time**	F(1,78)=17.2; p<0.001	
**Genotype**	APP/PS1>WT	F(1,78)=4.304; p<0.041

Cortical thickness

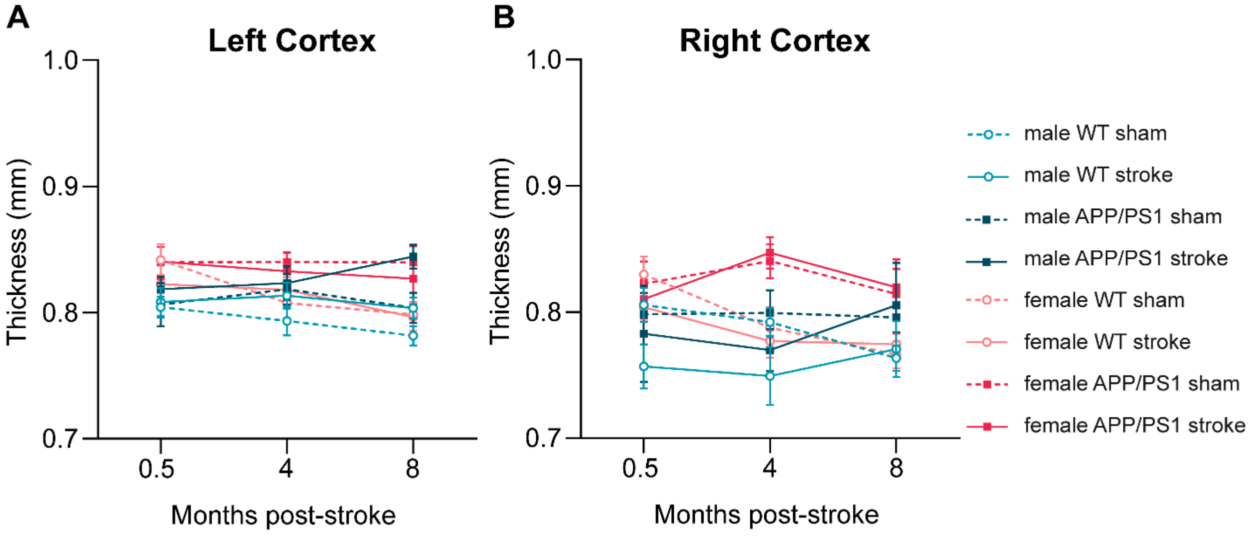

Supplementary Figure S7. Cortical thickness was measured in male and female wild-type (WT) and APP/PS1 mice at 0.5, 4, and 8 months post sham or stroke surgery. Measurements were conducted for both the left (A) and right (B) hemispheres. Data are presented as mean ± SEM.

Supplementary Table S16. Raw data of cortical thickness measurements, showing mean ± SEM and group size (n) in male and female wild-type (WT) and APP/PS1 mice, assessed at 0.5, 4, and 8 months following sham or stroke surgery.

		male WT sham		male WT stroke		male APP/PS1 sham		male APP/PS1 stroke		female WT sham		female WT stroke		female APP/PS1 sham		female APP/PS1 stroke	
		**mean ± SEM**	**n**	**mean ± SEM**	**n**	**mean ± SEM**	**n**	**mean ± SEM**	**n**	**mean ± SEM**	**n**	**mean ± SEM**	**n**	**mean ± SEM**	**n**	**mean ± SEM**	**n**
**Month 0.5**	**left CTX**	0.8 ± 0.01	14	0.81 ± 0.02	13	0.81 ± 0.02	7	0.82 ± 0.01	8	0.84 ± 0.01	14	0.82 ± 0.01	15	0.84 ± 0.01	10	0.84 ± 0.01	9
**Month 4**	**left CTX**	0.79 ± 0.01	13	0.82 ± 0.01	13	0.82 ± 0.01	7	0.82 ± 0.01	8	0.81 ± 0.01	15	0.82 ± 0.01	15	0.84 ± 0.01	10	0.83 ± 0.01	9
**Month 8**	**left CTX**	0.78 ± 0.01	13	0.8 ± 0.01	13	0.8 ± 0.01	6	0.84 ± 0.01	7	0.8 ± 0.01	15	0.8 ± 0.01	14	0.84 ± 0.01	10	0.83 ± 0.01	8
**Month 0.5**	**right CTX**	0.81 ± 0.01	14	0.8 ± 0.02	13	0.8 ± 0.02	7	0.78 ± 0.04	8	0.83 ± 0.01	14	0.8 ± 0.01	15	0.82 ± 0.02	10	0.8 ± 0.02	9
**Month 4**	**right CTX**	0.79 ± 0.01	13	0.8 ± 0.02	13	0.8 ± 0.02	7	0.77 ± 0.02	8	0.79 ± 0.02	15	0.78 ± 0.01	15	0.84 ± 0.01	10	0.82 ± 0.01	9
**Month 8**	**right CTX**	0.76 ± 0.01	13	0.8 ± 0.01	13	0.8 ± 0.01	6	0.81 ± 0.03	7	0.77 ± 0.01	15	0.77 ± 0.01	14	0.81 ± 0.02	10	0.82 ± 0.01	8

Supplementary Table S17. Results of the linear mixed model analysis for cortical thickness measurements, highlighting all significant effects of fixed factors (sex, genotype, surgery, time), and their respective interactions. The table specifies the direction of each effect, with numbers specifying the time point or time span where the effect was observed. F-values, degrees of freedom, and p-values are included for each effect.

	left CTX		right CTX	
	**Direction**	**p-value**	**Direction**	**p-value**
**Sex**	female>male	F(1,87)=8.08; p<0.006	female>male	F(1,87)=6.94; p<0.01
**Genotype**	APP/PS1>WT	F(1,87)=17.11; p<0.001	APP/PS1>WT	F(1,87)=10.32; p<0.002
**Surgery**			stroke<sham	F(1,86)=4.62; p<0.034

Supplementary Table S18. Results of the linear mixed model analysis for cortical thickness measurements. The table highlights all significant effects of fixed factors (sex, genotype, surgery, left vs right hemisphere) and their respective interactions per measurement time point. F-values, degrees of freedom, and p-values are included for each effect.

	Sex		Genotype		left vs right	
	**Direction**	**p-value**	**Direction**	**p-value**	**Direction**	**p-value**
**Month 0.5**	female>male	F(1,177)=15.02; p<0.001			right<left	F(1,177)=8.87; p<0.003
**Month 4**	female>male	F(1,174)=6.6; p<0.011	APP/PS1>WT	F(1,174)=13.59; p<0.001	right<left	F(1,174)=15.07; p<0.001
**Month 8**			APP/PS1>WT	F(1,171)=32.63; p<0.001	right<left	F(1,171)=14.09; p<0.001

Hippocampal Volume

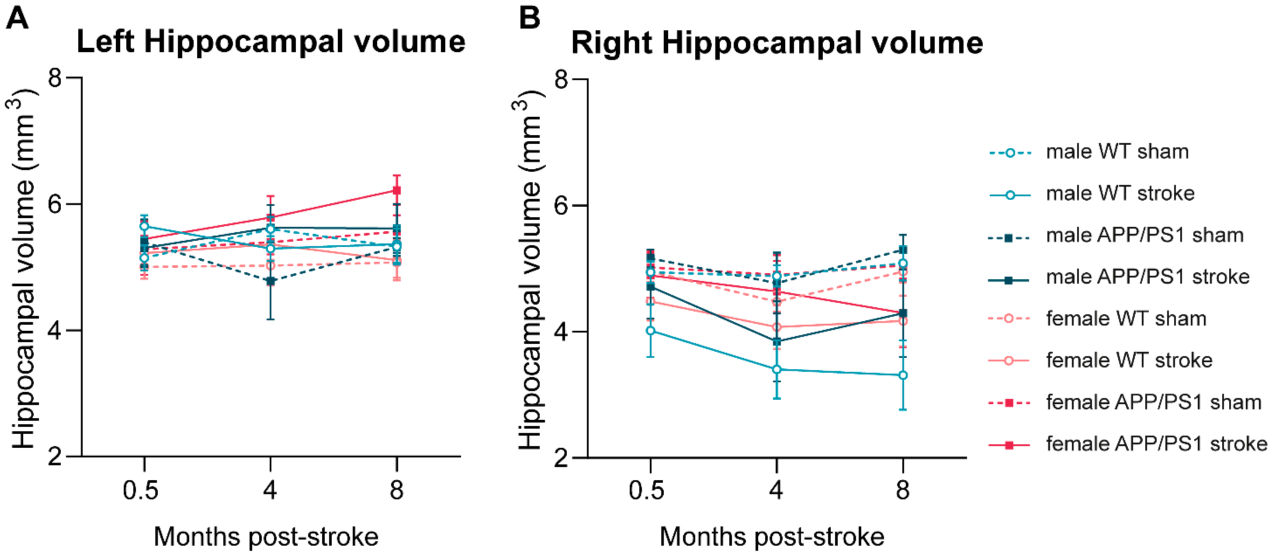

Supplementary Figure S8. Hippocampal volume was measured in male and female wild-type (WT) and APP/PS1 mice at 0.5, 4, and 8 months post sham or stroke surgery. Measurements were conducted for both the left (A) and right (B) hemispheres. Data are presented as mean ± SEM.

Supplementary Table S19. Raw data from hippocampal volume measurements displaying mean ± SEM and group size (n) of male and female wild-type (WT) and APP/PS1 mice at 0.5, 4, and 8 months post-stroke or sham surgery.

		male WT sham		male WT stroke		male APP/PS1 sham		male APP/PS1 stroke		female WT sham		female WT stroke		female APP/PS1 sham		female APP/PS1 stroke	
		**mean ± SEM**	**n**	**mean ± SEM**	**n**	**mean ± SEM**	**n**	**mean ± SEM**	**n**	**mean ± SEM**	**n**	**mean ± SEM**	**n**	**mean ± SEM**	**n**	**mean ± SEM**	**n**
**Month 0.5**	**left HIP**	5.15 ± 0.2	14	5.652 ± 0.173	13	5.396 ± 0.361	7	5.304 ± 0.304	8	5.003 ± 0.187	15	5.224 ± 0.235	15	5.283 ± 0.406	10	5.446 ± 0.278	9
**Month 0.5**	**right HIP**	4.945 ± 0.173	14	4.021 ± 0.416	13	5.165 ± 0.144	7	4.718 ± 0.509	8	4.956 ± 0.253	15	4.481 ± 0.304	15	5.019 ± 0.262	10	4.896 ± 0.353	9
**Month 4**	**left HIP**	5.607 ± 0.195	14	5.294 ± 0.201	13	4.784 ± 0.612	7	5.626 ± 0.363	8	5.024 ± 0.314	15	5.358 ± 0.248	15	5.396 ± 0.195	10	5.787 ± 0.34	9
**Month 4**	**right HIP**	4.883 ± 0.169	14	3.402 ± 0.464	13	4.774 ± 0.485	7	3.848 ± 0.634	8	4.477 ± 0.159	15	4.077 ± 0.348	15	4.904 ± 0.221	10	4.64 ± 0.573	9
**Month 8**	**left HIP**	5.325 ± 0.286	13	5.366 ± 0.296	13	5.319 ± 0.144	7	5.613 ± 0.387	7	5.076 ± 0.244	15	5.114 ± 0.321	15	5.565 ± 0.26	10	6.221 ± 0.237	8
**Month 8**	**right HIP**	5.086 ± 0.274	13	3.313 ± 0.55	13	5.302 ± 0.237	7	4.295 ± 0.699	7	4.953 ± 0.177	15	4.174 ± 0.401	15	5.05 ± 0.27	10	4.298 ± 0.545	8

Supplementary Table S20. Analysis of hippocampal volume in the left and right hemisphere using linear mixed model analysis, highlighting all significant effects of fixed factors (sex, genotype, surgery, time), and their respective interactions. The table specifies the direction of each effect, with numbers specifying the time point or time span where the effect was observed. F-values, degrees of freedom, and p-values are included for each effect.

	left HIP			right HIP		
	Direction		p-value	Direction		p-value
**Surgery**				stroke<sham		F(1,89)=12.45; p<0.001
**Time**				0.5→4	decreasing	F(2,177)=6.18; p<0.002
**Sex*Genotype**	female	APP/PS1>WT	F(1,87)=7.9; p<0.006			

Supplementary Table S21. Results of the linear mixed model analysis for hippocampal volume measurements. The table highlights all significant effects of fixed factors (sex, genotype, surgery, left vs right hemisphere) and their respective interactions per measurement time point. F-values, degrees of freedom, and p-values are included for each effect.

	Surgery*left vs right		
	Direction		p-value
**Month 0.5**	stroke	right<left	F(1,178)=21.13; p<0.001
	right	stroke<sham	F(1,178)=6.76; p<0.01
**Month 4**	sham	right<left	F(1,178)=4.63; p<0.033
	stroke	right<left	F(1,178)=40.55; p<0.001
	right	stroke<sham	F(1,178)=10.93; p<0.001
**Month 8**	stroke	right<left	F(1,172)=35.78; p<0.001
	right	stroke<sham	F(1,172)=19.52; p<0.001

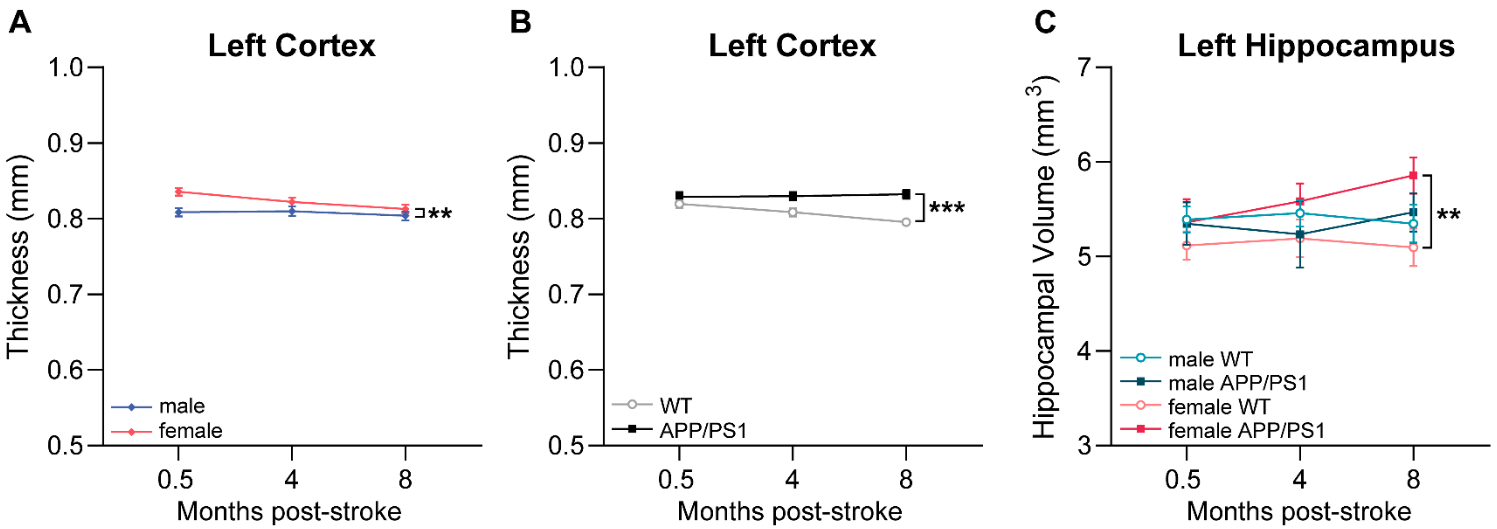

Supplementary Figure S9. Cortical thickness and hippocampal volume in male and female wild-type (WT) and APP/PS1 mice at 0.5, 4, and 8 months post-sham or stroke surgery. (A) Female mice generally had a thicker left cortex compared to male mice (male n=39-42, female n=47-49). (B) Similarly, APP/PS1 mice had a thicker left cortex compared to WT mice (WT n=55-56, APP/PS1 n=31-34). (C) In terms of hippocampal volume, among female mice, the APP/PS1 genotype displayed a higher left hippocampal volume than WT mice (male WT n=26-27, male APP/PS1 n=14-15, female WT n=30, female APP/PS1 n=18-19). Data are presented as mean ± SEM. Significance is denoted as $^{**}p < 0.01$, $^{***}p < 0.001$.

Cerebral blood flow

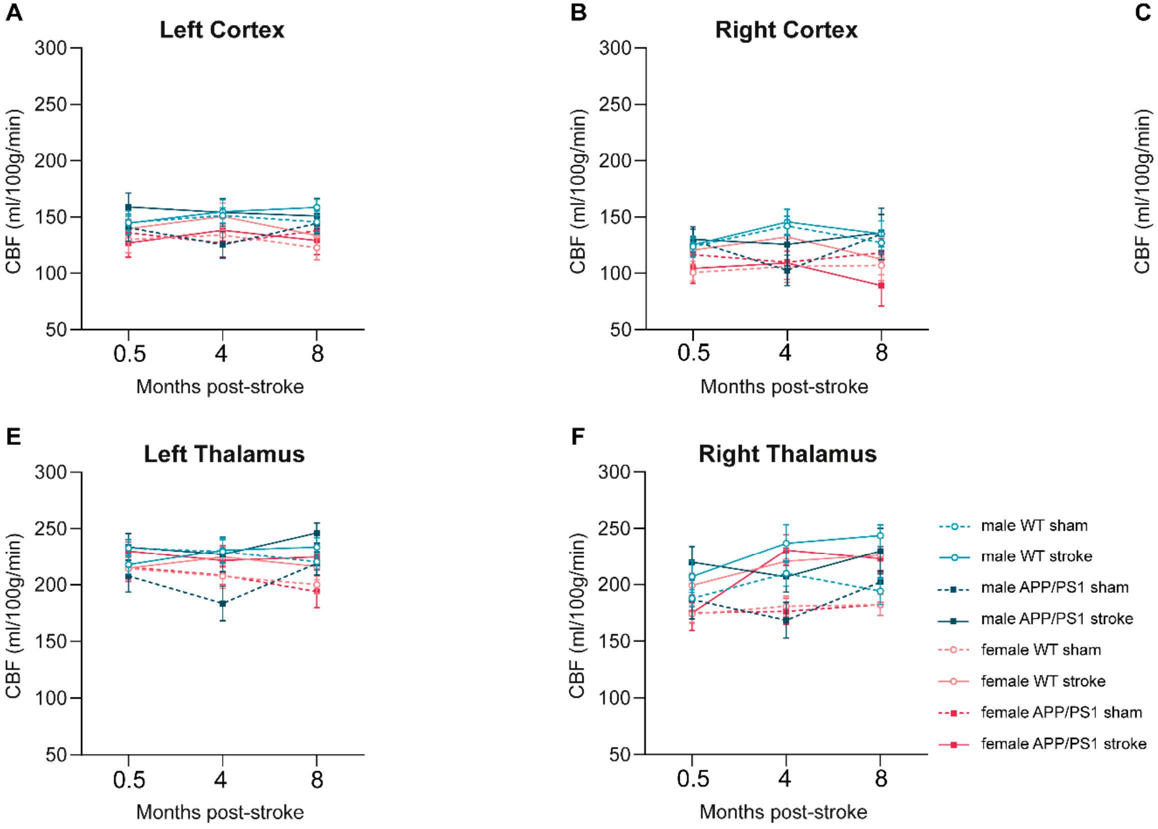

Supplementary Figure S10. Cerebral blood flow was measured in male and female wild-type (WT) and APP/PS1 mice at 0.5, 4, and 8 months post sham or stroke surgery. Measurements were conducted the in the (A) left cortex, (B) right cortex, (C) left hippocampus, (D) right hippocampus, (E) left thalamus, and (F) right thalamus. Data are presented as mean ± SEM.

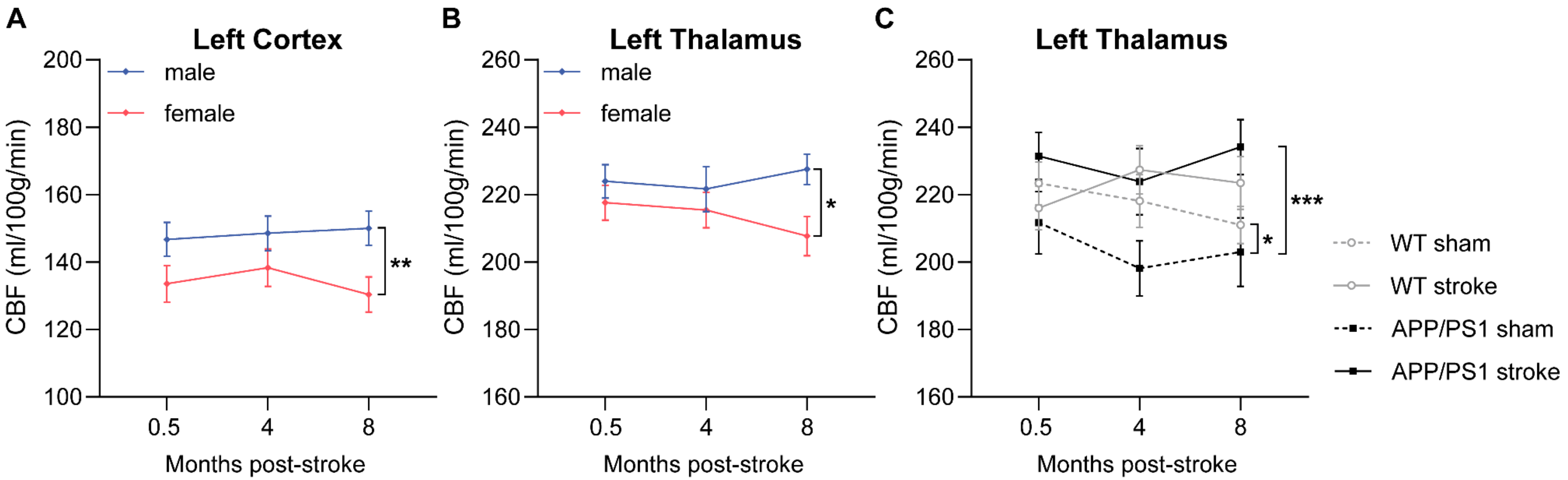

Supplementary Figure S11. Longitudinal analysis of cerebral blood flow in male and female wild-type (WT) and APP/PS1 mice at 0.5, 4, and 8 months post-stroke or sham surgery. (A) Female mice had overall lower CBF in the left cortex compared to male animals (male n=30-42, female n=35-49). (B) Similarly, female mice had overall lower CBF in the left thalamus compared to male animals (male n=30-42, female n=35-49). (C) In the left thalamus, APP/PS1 stroke mice exhibited higher CBF than APP/PS1 sham mice, and APP/PS1 sham mice had overall higher CBF compared to WT sham mice (WT sham n=24-29, WT stroke n=21-28, APP/PS1 sham n=11-17, APP/PS1 stroke n=9-17). Data are presented as mean ± SEM. Significance is denoted as *$p < 0.05$, **$p < 0.01$, ***$p < 0.001$.

Supplementary Table S22. Raw data from cerebral blood flow measurements displaying mean ± SEM and group size (n) of male and female wild-type (WT) and APP/PS1 mice at 0.5, 4, and 8 months post-stroke or sham surgery. Regions of interest include the cortex (CTX), hippocampus (HIP), and thalamus (TH) in both hemispheres.

		male WT sham		male WT stroke		male APP/PS1 sham		male APP/PS1 stroke		female WT sham		female WT stroke		female APP/PS1 sham		female APP/PS1 stroke	
		**mean ± SEM**	**n**	**mean ± SEM**	**n**	**mean ± SEM**	**n**	**mean ± SEM**	**n**	**mean ± SEM**	**n**	**mean ± SEM**	**n**	**mean ± SEM**	**n**	**mean ± SEM**	**n**
**Month 0.5**	**left CTX**	144.93 ± 10.7	14	144.53 ± 6.43	13	140.49 ± 12.03	7	158.86 ± 12.26	8	129.86 ± 11.75	14	139.74 ± 8.47	15	135.82 ± 11.14	8	126.9 ± 12.8	9
**Month 4**	**left CTX**	151.25 ± 6.91	14	154.71 ± 10.65	13	125.35 ± 11.91	7	154.03 ± 12.21	8	133.99 ± 9.87	15	150.43 ± 12.1	15	126.77 ± 12.62	10	138.24 ± 6.14	9
**Month 8**	**left CTX**	145.5 ± 9.59	13	158.57 ± 7.36	9	144.31 ± 4.88	4	150.91 ± 15.76	4	122.77 ± 10.88	11	133.58 ± 9.23	12	137.7 ± 9.66	7	129.17 ± 12.63	5
**Month 0.5**	**right CTX**	123.74 ± 9.42	14	125.17 ± 5.97	13	129.02 ± 10.15	7	130.13 ± 11	8	100.61 ± 7.75	14	120.61 ± 10.02	15	116.63 ± 9.52	8	104.27 ± 13.08	9
**Month 4**	**right CTX**	142.06 ± 8.7	14	145.67 ± 11.22	13	102.59 ± 13.55	7	125.73 ± 19.23	8	105.91 ± 14.17	15	132.29 ± 8.96	15	109.8 ± 9.76	10	109.2 ± 14.74	9
**Month 8**	**right CTX**	127.21 ± 9.56	13	134.89 ± 11.52	9	135.02 ± 22.95	4	136.02 ± 16.15	4	106.96 ± 13.57	11	112.51 ± 14	12	118.29 ± 12.53	7	89.06 ± 18.3	5
**Month 0.5**	**left HIP**	161.94 ± 10.57	14	166.43 ± 6.17	13	183.28 ± 10.95	7	172.67 ± 6.17	8	154.91 ± 11.78	14	156.23 ± 7.51	15	146.71 ± 12.01	8	168.93 ± 8.99	9
**Month 4**	**left HIP**	158.98 ± 12.3	14	169.84 ± 10.29	13	141.12 ± 15.48	7	160.88 ± 11.29	8	161.22 ± 7.03	15	150.12 ± 6.68	15	130.2 ± 8.77	10	174.86 ± 9.67	9
**Month 8**	**left HIP**	151.26 ± 9.68	13	142.29 ± 13.02	9	163.75 ± 18.43	4	165.92 ± 23	4	149.37 ± 12.46	11	149.21 ± 10.47	12	113.31 ± 10	7	149.22 ± 15.18	5
**Month 0.5**	**right HIP**	95.73 ± 8.48	14	106.02 ± 6.99	10	99.25 ± 13.74	7	110 ± 5.84	8	78.8 ± 10.68	13	100.18 ± 12.07	14	96.41 ± 9.44	7	91.87 ± 8.88	9
**Month 4**	**right HIP**	108.94 ± 12.05	14	108.29 ± 10.37	9	84.75 ± 13.04	7	114.84 ± 14.99	8	100.76 ± 11.97	15	107.58 ± 7.33	11	88.94 ± 10.52	10	103.2 ± 16.39	8
**Month 8**	**right HIP**	103.23 ± 11.28	13	93.15 ± 12.22	5	129.73 ± 15.09	4	126.13 ± 21.58	4	112.39 ± 13.89	11	106.15 ± 12.98	8	97.92 ± 10.23	7	90.93 ± 6.8	5
**Month 0.5**	**left TH**	232.5 ± 7.64	14	217.78 ± 8.31	13	207.53 ± 14.28	7	233.39 ± 12.02	8	214.27 ± 9.86	14	214.56 ± 9.96	15	215.35 ± 12.75	8	229.74 ± 8.44	9
**Month 4**	**left TH**	229.31 ± 10.75	14	230.78 ± 11.21	13	183.54 ± 15.32	7	226.88 ± 15.46	8	207.63 ± 10.99	15	224.44 ± 9.43	15	208.31 ± 8.09	10	221.26 ± 13.36	9
**Month 8**	**left TH**	220.34 ± 6.98	13	233.4 ± 8.58	9	218.91 ± 10.48	4	246.1 ± 8.9	4	199.96 ± 7.68	11	216 ± 11.83	12	193.84 ± 14.19	7	224.48 ± 11.95	5
**Month 0.5**	**right TH**	187.62 ± 7.77	14	206.96 ± 14.08	13	186.63 ± 17.21	7	219.62 ± 14.07	8	173.98 ± 7.46	14	199.06 ± 10.64	15	174.95 ± 15.77	8	174.81 ± 8.98	9
**Month 4**	**right TH**	209.71 ± 11.13	14	236.6 ± 16.74	13	168.45 ± 15.67	7	206.81 ± 13.54	8	180.72 ± 8.94	15	220.41 ± 10.61	15	176.37 ± 11.56	10	230.51 ± 13.8	9
**Month 8**	**right TH**	194.16 ± 11.86	13	243.49 ± 9.69	9	202.05 ± 9.38	4	229.7 ± 20.48	4	181.99 ± 9.33	11	226.05 ± 15.46	12	182.29 ± 9.86	7	222.77 ± 10.95	5

Supplementary Table S23. Analysis of cerebral blood flow using linear mixed model analysis, highlighting all significant effects of fixed factors (sex, genotype, surgery, time) and their respective interactions. Regions of interest include the cortex (CTX), hippocampus (HIP), and thalamus (TH) in both hemispheres. The table specifies the direction of each effect, with numbers specifying the time point or time span where the effect was observed. F-values, degrees of freedom, and p-values are included for each effect.

	left CTX		right CTX		left HIP		right HIP		left TH		right TH	
	Direction	p-value	Direction	p-value	Direction	p-value	Direction	p-value	Direction	p-value	Direction	p-value
**Sex**	female<male	F(1,90)=9.13; p<0.003	female<male	F(1,91)=9.43; p<0.003					female<male	F(1,80)=5.39; p<0.023		
**Surgery**											stroke>sham	F(1,85)=26.74; p<0.001
**Time**					0.5→8 decreasing	F(2,172)=4.49; p<0.01						
**Surgery*Genotype**									sham APP/PS1<WT	F(1,83)=4.56; p<0.036		
									APP/PS1 stroke>sham	F(1,89)=12.18; p<0.001		
**Sex*Genotype*Time**											4 WT female<male	F(1,204)=4.61; p<0.033
											0.5 APP/PS1 female<male	F(1,208)=4.24; p<0.041
											4 male APP/PS1<WT	F(1,204)=7.61; p<0.006
											0.5→4 male WT increasing	F(2,146)=3.95; p<0.019
											0.5→4 female APP/PS1 increasing	F(2,150)=3.78; p<0.039

Supplementary Table S24. Results of the linear mixed model analysis of cerebral blood flow measurements. The table highlights all significant effects of fixed factors (sex, genotype, surgery, left vs right hemisphere) and their respective interactions per measurement time point. F-values, degrees of freedom, and p-values are included for each effect.

	ROI	Sex		Genotype		Surgery		Left vs right		Sex*Genotype	
		Direction	p-value	Direction	p-value	Direction	p-value	Direction	p-value	Direction	p-value
**Month 0.5**	**CTX**	female<male	F(1,173)=8.33; p<0.004					right<left	F(1,173)=18.96; p<0.001		
	**HIP**	female<male	F(1,167)=5.32; p<0.022					right<left	F(1,167)=177.46; p<0		
	**TH**							right<left	F(1,172)=30.33; p<0.001		
**Month 4**	**CTX**			APP/PS1<WT	F(1,178)=6.51; p<0.012			right<left	F(1,178)=11.15; p<0.001		
	**HIP**					stroke>sham	F(1,168)=6.59; p<0.011	right<left	F(1,168)=95.76; p<0.001		
	**TH**					stroke>sham	F(1,176)=20.29; p<0.001				
**Month 8**	**CTX**	female<male	F(1,127)=12.41; p<0.001					right<left	F(1,127)=11.1; p<0.001		
	**HIP**							right<left	F(1,117)=38.52; p<0.001	APP/PS1 female<male	F(1,117)=9.01; p<0.003
										male APP/PS1>WT	F(1,117)=4.31; p<0.04
	**TH**	female<male	F(1,127)=8.67; p<0.004			stroke>sham	F(1,127)=27.83; p<0.001				

Diffusion tensor imaging – Fractional anisotropy

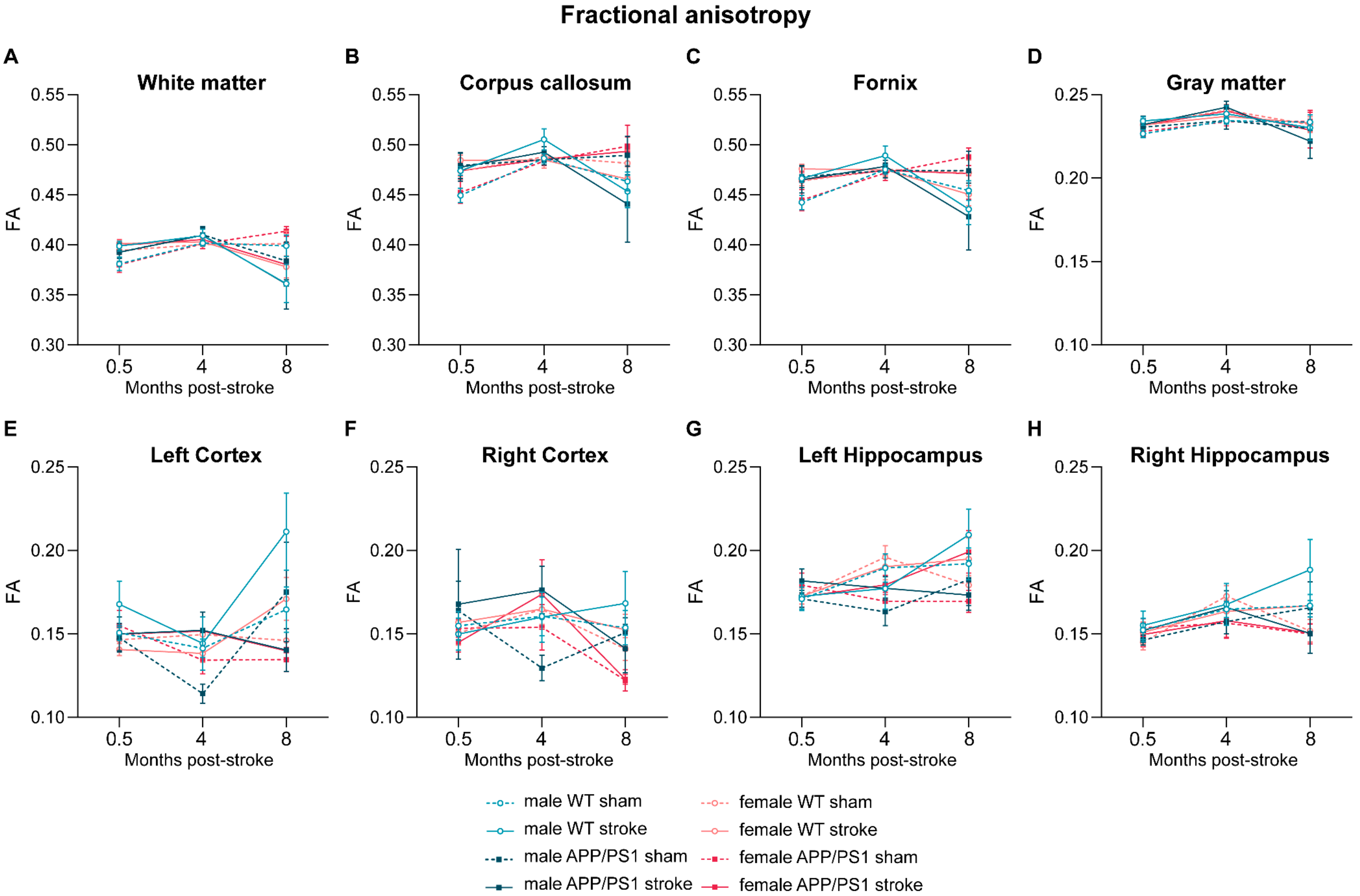

Supplementary Figure S12. Fractional anisotropy was assessed via diffusion tensor imaging in male and female wild-type (WT) and APP/PS1 mice at 0.5, 4, and 8 months post-sham or stroke surgery. Measurements were conducted in the (A) white matter, (B) corpus callosum, (C) fornix, (D) grey matter, (E) left cortex, (F) right cortex, (G) left hippocampus, and (H) right hippocampus. Data are presented as mean ± SEM.

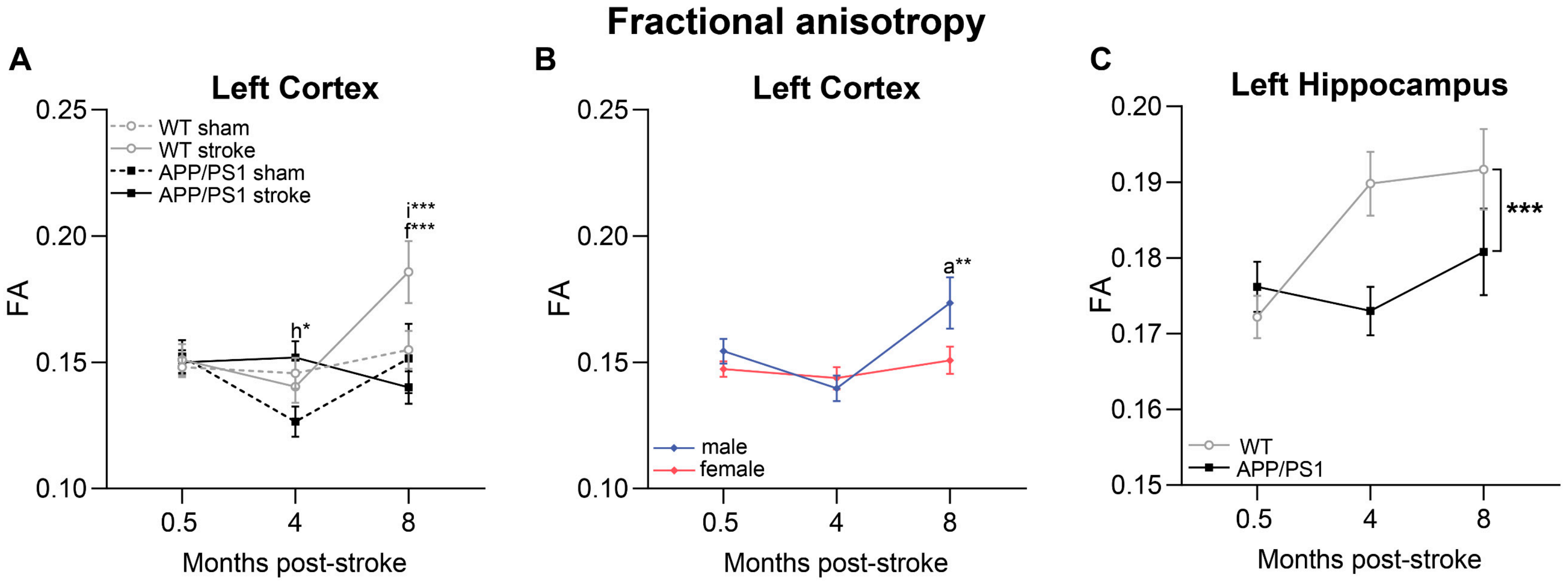

Supplementary Figure S13. Longitudinal diffusion tensor imaging analysis. A comprehensive representation of fractional anisotropy (FA) changes in male and female wild-type (WT) and APP/PS1 mice at 0.5, 4, and 8 months post-sham or stroke surgery. (A) In the left cortex, several genotype and surgery-related differences were observed. FA in APP/PS1 sham mice decreased from 0.5 to 4 months. In contrast, in WT stroke mice, FA increased from 0.5 to 8 months as well as from 4 to 8 months. At 4 months post-surgery, APP/PS1 stroke mice showed higher FA than APP/PS1 sham mice (h: APP/PS1 sham vs APP/PS1 stroke). At 8 months post-surgery, WT stroke mice showed higher FA compared to WT sham mice (f: WT sham vs WT stroke), while APP/PS1 stroke mice had lower FA than WT stroke mice (i: WT stroke vs APP/PS1 stroke); (WT sham n=23-27, WT stroke n=19-21, APP/PS1 sham n=12-16, APP/PS1 stroke n=11-16). (B) In male mice, FA increased in the left cortex from 4 to 8 months after surgery. At 8 months post-surgery, female mice exhibited lower FA in the left cortex compared to male mice (male n=29-31, female n=38-46). (C) Over time, APP/PS1 mice showed higher FA in the left hippocampus than WT mice (WT n=14-17, APP/PS1 n=23-32). Data are presented as mean ± SEM. Significance is denoted as *p < 0.05, **p < 0.01, ***p < 0.001.

Supplementary Table S25. Raw data of fractional anisotropy displaying mean ± SEM and group size (n) of male and female wild-type (WT) and APP/PS1 mice at 0.5, 4, and 8 months post-stroke or sham surgery. Regions of interest include white matter (WM), grey matter (GM), corpus callosum (CC), fornix (FX), left and right cortex (CTX), and left and right hippocampus (HIP).

		male WT sham		male WT stroke		male APP/PS1 sham		male APP/PS1 stroke		female WT sham		female WT stroke		female APP/PS1 sham		female APP/PS1 stroke	
		**mean ± SEM**	**n**	**mean ± SEM**	**n**	**mean ± SEM**	**n**	**mean ± SEM**	**n**	**mean ± SEM**	**n**	**mean ± SEM**	**n**	**mean ± SEM**	**n**	**mean ± SEM**	**n**
**Month 0.5**	**WM**	0.381 ± 0.007	9	0.399 ± 0.005	8	0.393 ± 0.006	6	0.393 ± 0.009	7	0.394 ± 0.004	14	0.401 ± 0.004	13	0.38 ± 0.008	10	0.399 ± 0.006	9
**Month 4**	**WM**	0.402 ± 0.003	13	0.409 ± 0.006	7	0.41 ± 0.009	5	0.41 ± 0.008	6	0.401 ± 0.002	14	0.403 ± 0.003	13	0.401 ± 0.005	8	0.405 ± 0.003	8
**Month 8**	**WM**	0.399 ± 0.011	12	0.361 ± 0.019	7	0.384 ± 0.019	5	0.361 ± 0.025	5	0.401 ± 0.013	13	0.378 ± 0.011	12	0.414 ± 0.005	7	0.38 ± 0.019	6
**Month 0.5**	**GM**	0.225 ± 0.003	9	0.235 ± 0.003	8	0.231 ± 0.005	6	0.231 ± 0.004	7	0.231 ± 0.003	14	0.232 ± 0.003	13	0.228 ± 0.003	10	0.232 ± 0.003	9
**Month 4**	**GM**	0.234 ± 0.002	13	0.238 ± 0.005	7	0.234 ± 0.005	5	0.242 ± 0.004	6	0.241 ± 0.003	14	0.237 ± 0.002	13	0.234 ± 0.003	8	0.24 ± 0.003	8
**Month 8**	**GM**	0.234 ± 0.004	12	0.23 ± 0.008	7	0.229 ± 0.01	5	0.222 ± 0.01	5	0.233 ± 0.005	13	0.233 ± 0.005	12	0.24 ± 0.004	7	0.229 ± 0.011	6
**Month 0.5**	**CC**	0.449 ± 0.007	9	0.474 ± 0.004	8	0.479 ± 0.013	6	0.478 ± 0.014	7	0.474 ± 0.005	14	0.485 ± 0.005	13	0.452 ± 0.011	10	0.474 ± 0.008	9
**Month 4**	**CC**	0.486 ± 0.004	13	0.505 ± 0.011	7	0.485 ± 0.005	5	0.493 ± 0.005	6	0.488 ± 0.006	14	0.485 ± 0.008	13	0.484 ± 0.007	8	0.485 ± 0.006	8
**Month 8**	**CC**	0.463 ± 0.01	12	0.453 ± 0.016	7	0.489 ± 0.019	5	0.441 ± 0.038	5	0.481 ± 0.013	13	0.466 ± 0.012	12	0.498 ± 0.009	7	0.493 ± 0.026	6
**Month 0.5**	**FX**	0.442 ± 0.007	9	0.466 ± 0.004	8	0.468 ± 0.011	6	0.465 ± 0.013	7	0.464 ± 0.005	14	0.476 ± 0.005	13	0.445 ± 0.011	10	0.465 ± 0.008	9
**Month 4**	**FX**	0.475 ± 0.004	13	0.489 ± 0.01	7	0.474 ± 0.007	5	0.478 ± 0.006	6	0.475 ± 0.005	14	0.475 ± 0.007	13	0.472 ± 0.008	8	0.474 ± 0.006	8
**Month 8**	**FX**	0.454 ± 0.01	12	0.436 ± 0.016	7	0.474 ± 0.019	5	0.428 ± 0.033	5	0.472 ± 0.013	13	0.45 ± 0.011	12	0.488 ± 0.009	7	0.471 ± 0.026	6
**Month 0.5**	**left CTX**	0.151 ± 0.005	9	0.168 ± 0.014	8	0.148 ± 0.009	6	0.15 ± 0.01	7	0.146 ± 0.006	14	0.141 ± 0.004	13	0.155 ± 0.009	10	0.15 ± 0.004	9
**Month 4**	**left CTX**	0.141 ± 0.005	13	0.144 ± 0.016	7	0.114 ± 0.006	5	0.152 ± 0.011	6	0.15 ± 0.011	14	0.138 ± 0.005	13	0.134 ± 0.008	8	0.152 ± 0.008	8
**Month 8**	**left CTX**	0.165 ± 0.014	12	0.211 ± 0.023	7	0.175 ± 0.03	5	0.14 ± 0.013	5	0.146 ± 0.007	13	0.171 ± 0.013	12	0.135 ± 0.007	7	0.14 ± 0.006	6
**Month 0.5**	**right CTX**	0.155 ± 0.009	9	0.15 ± 0.01	8	0.164 ± 0.017	6	0.168 ± 0.033	7	0.151 ± 0.007	14	0.157 ± 0.008	13	0.153 ± 0.005	10	0.145 ± 0.006	9
**Month 4**	**right CTX**	0.161 ± 0.012	13	0.16 ± 0.015	7	0.13 ± 0.008	5	0.176 ± 0.014	6	0.165 ± 0.009	14	0.165 ± 0.012	13	0.154 ± 0.014	8	0.174 ± 0.021	8
**Month 8**	**right CTX**	0.154 ± 0.01	12	0.168 ± 0.019	7	0.151 ± 0.009	5	0.141 ± 0.015	5	0.141 ± 0.007	13	0.152 ± 0.009	12	0.122 ± 0.006	7	0.123 ± 0.003	6
**Month 0.5**	**left HIP**	0.171 ± 0.007	9	0.172 ± 0.008	8	0.171 ± 0.005	6	0.182 ± 0.007	7	0.173 ± 0.005	14	0.173 ± 0.005	13	0.179 ± 0.007	10	0.172 ± 0.006	9
**Month 4**	**left HIP**	0.189 ± 0.009	13	0.177 ± 0.012	7	0.163 ± 0.008	5	0.177 ± 0.007	6	0.196 ± 0.007	14	0.19 ± 0.008	13	0.17 ± 0.005	8	0.179 ± 0.006	8
**Month 8**	**left HIP**	0.192 ± 0.01	12	0.209 ± 0.015	7	0.183 ± 0.016	5	0.173 ± 0.009	5	0.179 ± 0.006	13	0.195 ± 0.013	12	0.169 ± 0.007	7	0.199 ± 0.013	6
**Month 0.5**	**right HIP**	0.152 ± 0.007	9	0.155 ± 0.009	8	0.146 ± 0.003	6	0.152 ± 0.004	7	0.145 ± 0.005	14	0.151 ± 0.006	13	0.154 ± 0.006	10	0.15 ± 0.007	9
**Month 4**	**right HIP**	0.165 ± 0.005	13	0.168 ± 0.013	7	0.157 ± 0.007	5	0.166 ± 0.01	6	0.173 ± 0.006	14	0.164 ± 0.007	13	0.156 ± 0.008	8	0.158 ± 0.01	8
**Month 8**	**right HIP**	0.167 ± 0.007	12	0.188 ± 0.018	7	0.166 ± 0.016	5	0.15 ± 0.012	5	0.152 ± 0.007	13	0.167 ± 0.007	12	0.15 ± 0.006	7	0.151 ± 0.006	6

Supplementary Table S26. Analysis of fractional anisotropy using linear mixed model analysis, highlighting all significant effects of fixed factors (sex, genotype, surgery, time) and their respective interactions. Regions of interest include white matter (WM), grey matter (GM), corpus callosum (CC), fornix (FX), left and right cortex (CTX), and left and right hippocampus (HIP). The table specifies the direction of each effect, with numbers indicating the time point or time span where the effect was observed. F-values, degrees of freedom, and p-values are included for each effect.

	WM		GM		CC		F		left CTX		right CTX		left HIP		right HIP	
	Direction	p-value	Direction	p-value	Direction	p-value	Direction	p-value	Direction	p-value	Direction	p-value	Direction	p-value	Direction	p-value
Genotype													APP/PS1<WT	F(1,78)=4.22; p<0.043		
Time	4→8 decreasing	F(2,162)=6.31; p<0.002	0.5→4 increasing	F(2,150)=6.02; p<0.003	0.5→4 increasing	F(2,152)=6.67; p<0.004	0.5→4 increasing	F(2,155)=5.56; p<0.024			4→8 decreasing	F(2,157)=3.73; p<0.021	0.5→8 increasing	F(2,149)=5.07; p<0.007	0.5→4 increasing	F(2,141)=6.42; p<0.003
			4→8 decreasing	F(2,157)=6.02; p<0.038	4→8 decreasing	F(2,158)=6.67; p<0.009	4→8 decreasing	F(2,162)=5.56; p<0.008							0.5→8 increasing	F(2,147)=6.42; p<0.03
Sex*Time									8 female<male	F(1,200)=10.07; p<0.002						
									4→8 increasing: male	F(2,145)=9.38; p<0.001						
Genotype*Surgery*Time									8 stroke APP/PS1<WT	F(1,201)=16.35; p<0.001						
									8 WT stroke>sham	F(1,199)=12.08; p<0.001						
									4 APP/PS1 stroke>sham	F(1,199)=4.62; p<0.033						
									0.5→8 WT stroke increasing	F(2,140)=14.47; p<0.001						
									4→8 WT stroke increasing	F(2,142)=14.47; p<0.001						
									0.5→4 APP/PS1 sham decreasing	F(2,140)=3.75; p<0.048						

Supplementary Table S27. Results of the linear mixed model analysis of fractional anisotropy. The table highlights all significant effects of fixed factors (sex, genotype, surgery, left vs. right hemisphere) and their respective interactions per measurement time point. F-values, degrees of freedom, and p-values are included for each effect.

		Sex		Genotype		left vs right		Genotype*Surgery		
		**Direction**	**p-value**	**Direction**	**p-value**	**Direction**	**p-value**	**Direction**		**p-value**
**Month 0.5**	**HIP**					right<left	F(1,150)=59.67; p<0.001			
**Month 4**	**CTX**					right>left	F(1,143)=12.64; p<0.001	sham	APP/PS1<WT	F(1,143)=5.29; p<0.023
								APP/PS1	stroke>sham	F(1,143)=9.07; p<0.003
	**HIP**			APP/PS1<WT	F(1,145)=8.89; p<0.003	right<left	F(1,145)=23.55; p<0.001			
**Month 8**	**CTX**	female<male	F(1,128)=11.58; p<0.001	APP/PS1<WT	F(1,128)=11.88; p<0.001					
	**HIP**			APP/PS1<WT	F(1,131)=4.38; p<0.038	right<left	F(1,131)=25.37; p<0.001			

Diffusion tensor imaging – Mean diffusivity

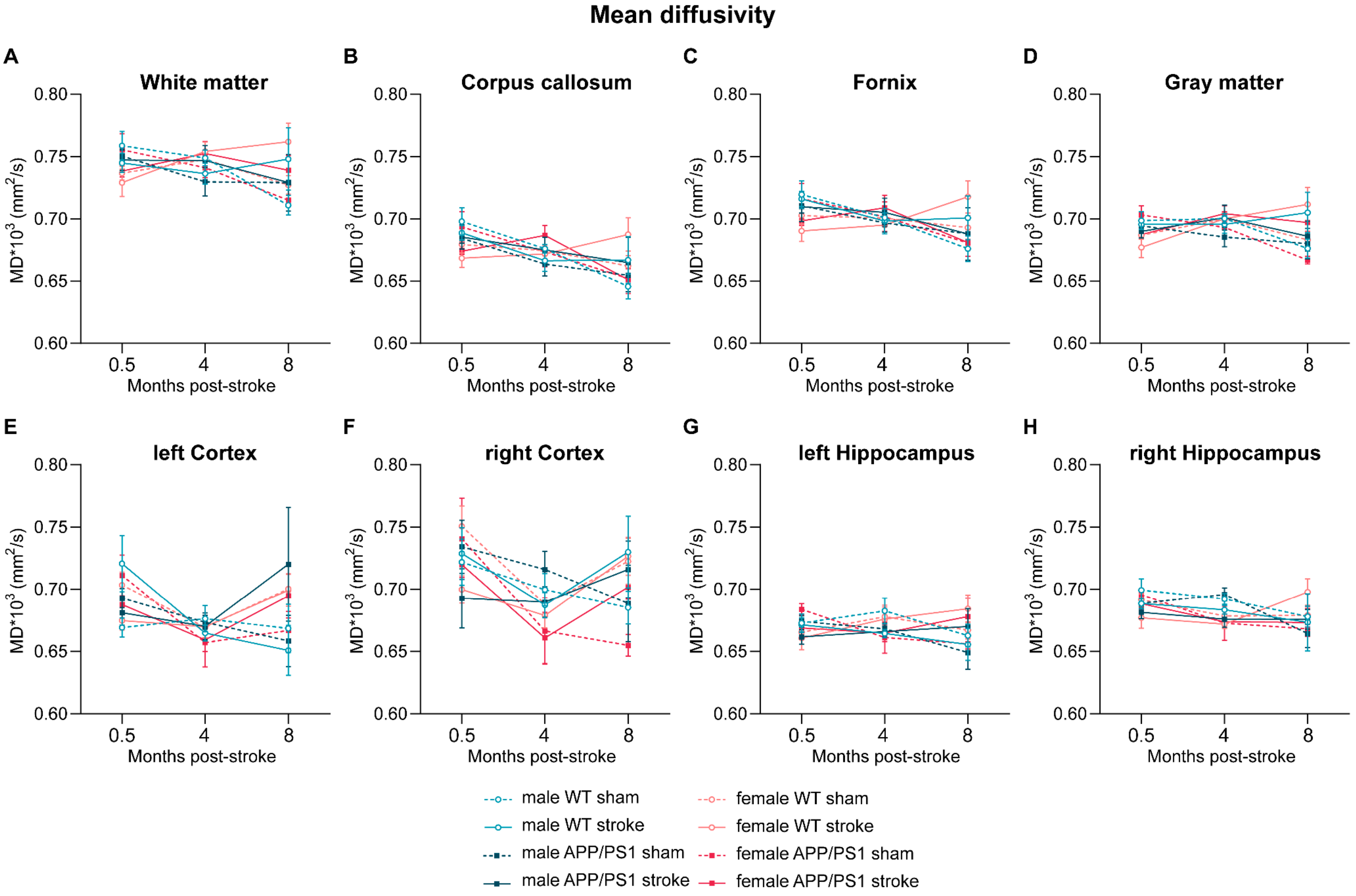

Supplementary Figure S14. Mean diffusivity was assessed via diffusion tensor imaging in male and female wild-type (WT) and APP/PS1 mice at 0.5, 4, and 8 months post-sham or stroke surgery. Measurements were conducted in the (A) white matter, (B) corpus callosum, (C) fornix, (D) grey matter, (E) left cortex, (F) right cortex, (G) left hippocampus, and (H) right hippocampus. Data are presented as mean ± SEM.

Supplementary Table S28. Raw data of mean diffusivity displaying mean ± SEM and group size (n) of male and female wild-type (WT) and APP/PS1 mice at 0.5, 4, and 8 months post-stroke or sham surgery. Regions of interest include white matter (WM), grey matter (GM), corpus callosum (CC), fornix (FX), left and right cortex (CTX), and left and right hippocampus (HIP).

		male WT sham		male WT stroke		male APP/PS1 sham		male APP/PS1 stroke		female WT sham		female WT stroke		female APP/PS1 sham		female APP/PS1 stroke	
		**mean ± SEM**	**n**	**mean ± SEM**	**n**	**mean ± SEM**	**n**	**mean ± SEM**	**n**	**mean ± SEM**	**n**	**mean ± SEM**	**n**	**mean ± SEM**	**n**	**mean ± SEM**	**n**
**Month 0.5**	**WM**	0.759 ± 0.012	9	0.745 ± 0.008	8	0.75 ± 0.006	6	0.747 ± 0.008	7	0.737 ± 0.005	14	0.729 ± 0.011	13	0.755 ± 0.013	10	0.738 ± 0.005	9
**Month 4**	**WM**	0.749 ± 0.006	13	0.736 ± 0.008	7	0.73 ± 0.011	5	0.747 ± 0.012	6	0.748 ± 0.008	14	0.754 ± 0.008	13	0.741 ± 0.008	8	0.752 ± 0.01	8
**Month 8**	**WM**	0.711 ± 0.008	12	0.748 ± 0.025	7	0.729 ± 0.023	5	0.729 ± 0.018	5	0.727 ± 0.007	13	0.762 ± 0.015	12	0.715 ± 0.004	7	0.739 ± 0.012	6
**Month 0.5**	**GM**	0.698 ± 0.007	9	0.696 ± 0.004	8	0.694 ± 0.004	6	0.69 ± 0.005	7	0.687 ± 0.003	14	0.677 ± 0.008	13	0.703 ± 0.007	10	0.687 ± 0.003	9
**Month 4**	**GM**	0.7 ± 0.004	13	0.695 ± 0.007	7	0.685 ± 0.008	5	0.701 ± 0.01	6	0.7 ± 0.005	14	0.7 ± 0.005	13	0.693 ± 0.008	8	0.704 ± 0.006	8
**Month 8**	**GM**	0.676 ± 0.008	12	0.705 ± 0.016	7	0.68 ± 0.012	5	0.686 ± 0.012	5	0.683 ± 0.006	13	0.712 ± 0.014	12	0.667 ± 0.003	7	0.697 ± 0.007	6
**Month 0.5**	**CC**	0.698 ± 0.011	9	0.689 ± 0.006	8	0.684 ± 0.004	6	0.685 ± 0.01	7	0.679 ± 0.007	14	0.668 ± 0.007	13	0.693 ± 0.012	10	0.674 ± 0.002	9
**Month 4**	**CC**	0.676 ± 0.003	13	0.666 ± 0.009	7	0.664 ± 0.01	5	0.675 ± 0.01	6	0.674 ± 0.004	14	0.672 ± 0.005	13	0.673 ± 0.012	8	0.687 ± 0.008	8
**Month 8**	**CC**	0.646 ± 0.01	12	0.667 ± 0.019	7	0.655 ± 0.013	5	0.665 ± 0.02	5	0.662 ± 0.009	13	0.687 ± 0.013	12	0.652 ± 0.008	7	0.651 ± 0.011	6
**Month 0.5**	**FX**	0.72 ± 0.011	9	0.716 ± 0.006	8	0.711 ± 0.006	6	0.71 ± 0.012	7	0.703 ± 0.007	14	0.69 ± 0.009	13	0.716 ± 0.012	10	0.698 ± 0.004	9
**Month 4**	**FX**	0.701 ± 0.004	13	0.698 ± 0.01	7	0.697 ± 0.007	5	0.705 ± 0.011	6	0.701 ± 0.006	14	0.695 ± 0.006	13	0.702 ± 0.012	8	0.709 ± 0.01	8
**Month 8**	**FX**	0.676 ± 0.01	12	0.701 ± 0.017	7	0.688 ± 0.013	5	0.688 ± 0.021	5	0.693 ± 0.01	13	0.718 ± 0.013	12	0.681 ± 0.011	7	0.681 ± 0.011	6
**Month 0.5**	**left CTX**	0.669 ± 0.008	9	0.721 ± 0.023	8	0.693 ± 0.008	6	0.681 ± 0.01	7	0.703 ± 0.011	14	0.675 ± 0.008	13	0.711 ± 0.016	10	0.687 ± 0.007	9
**Month 4**	**left CTX**	0.676 ± 0.011	13	0.665 ± 0.007	7	0.673 ± 0.006	5	0.67 ± 0.011	6	0.669 ± 0.008	14	0.67 ± 0.006	13	0.657 ± 0.02	8	0.659 ± 0.009	8
**Month 8**	**left CTX**	0.668 ± 0.019	12	0.651 ± 0.02	7	0.658 ± 0.02	5	0.72 ± 0.046	5	0.7 ± 0.012	13	0.699 ± 0.013	12	0.667 ± 0.015	7	0.694 ± 0.018	6
**Month 0.5**	**right CTX**	0.722 ± 0.019	9	0.729 ± 0.021	8	0.734 ± 0.021	6	0.693 ± 0.024	7	0.75 ± 0.016	14	0.7 ± 0.011	13	0.74 ± 0.033	10	0.72 ± 0.01	9
**Month 4**	**right CTX**	0.7 ± 0.013	13	0.687 ± 0.01	7	0.716 ± 0.015	5	0.689 ± 0.012	6	0.688 ± 0.01	14	0.679 ± 0.01	13	0.666 ± 0.027	8	0.661 ± 0.021	8
**Month 8**	**right CTX**	0.685 ± 0.013	12	0.73 ± 0.029	7	0.688 ± 0.031	5	0.716 ± 0.023	5	0.722 ± 0.019	13	0.726 ± 0.015	12	0.655 ± 0.009	7	0.701 ± 0.01	6
**Month 0.5**	**left HIP**	0.673 ± 0.007	9	0.671 ± 0.006	8	0.674 ± 0.005	6	0.662 ± 0.006	7	0.665 ± 0.004	14	0.66 ± 0.009	13	0.684 ± 0.005	10	0.669 ± 0.005	9
**Month 4**	**left HIP**	0.682 ± 0.01	13	0.664 ± 0.003	7	0.668 ± 0.004	5	0.666 ± 0.004	6	0.678 ± 0.008	14	0.676 ± 0.011	13	0.661 ± 0.013	8	0.665 ± 0.007	8
**Month 8**	**left HIP**	0.663 ± 0.009	12	0.655 ± 0.013	7	0.649 ± 0.013	5	0.67 ± 0.015	5	0.667 ± 0.007	13	0.684 ± 0.011	12	0.656 ± 0.004	7	0.678 ± 0.015	6
**Month 0.5**	**right HIP**	0.699 ± 0.009	9	0.688 ± 0.004	8	0.689 ± 0.004	6	0.681 ± 0.005	7	0.691 ± 0.005	14	0.677 ± 0.008	13	0.696 ± 0.005	10	0.688 ± 0.005	9
**Month 4**	**right HIP**	0.692 ± 0.004	13	0.683 ± 0.003	7	0.696 ± 0.005	5	0.676 ± 0.007	6	0.678 ± 0.005	14	0.672 ± 0.004	13	0.673 ± 0.014	8	0.674 ± 0.005	8
**Month 8**	**right HIP**	0.678 ± 0.008	12	0.673 ± 0.023	7	0.664 ± 0.011	5	0.676 ± 0.008	5	0.679 ± 0.007	13	0.698 ± 0.011	12	0.668 ± 0.005	7	0.673 ± 0.011	6

Supplementary Table S29. Analysis of mean diffusivity using linear mixed model analysis, highlighting all significant effects of fixed factors (sex, genotype, surgery, time) and their respective interactions. Regions of interest include white matter (WM), grey matter (GM), corpus callosum (CC), fornix (FX), left and right cortex (CTX), and left and right hippocampus (HIP). The table specifies the direction of each effect, with numbers indicating the time point or time span where the effect was observed. F-values, degrees of freedom, and p-values are included for each effect.

	**CC**			**FX**			**left CTX**			**right CTX**		
	**Direction**		**p-value**	**Direction**		**p-value**	**Direction**		**p-value**	**Direction**		**p-value**
**Time**	0.5→8	decreasing	F(2,149)=8.78; p<0.001	0.5→8	decreasing	F(2,148)=3.58; p<0.025	0.5→4	decreasing	F(2,140)=6.61; p<0.001	0.5→4	decreasing	F(2,143)=9.82; p<0.001

Supplementary Table S30. Results of the linear mixed model analysis of mean diffusivity. The table highlights all significant effects of fixed factors (sex, genotype, surgery, left vs. right hemisphere) and their respective interactions per measurement time point. F-values, degrees of freedom, and p-values are included for each effect.

		**Surgery**		**left vs right**		**Genotype*Sex**		
		**Direction**	**p-value**	**Direction**	**p-value**	**Direction**		**p-value**
**Month 0.5**	**CTX**			right>left	F(1,143)=15.44; p<0.001			
	**HIP**	stroke<sham	F(1,146)=8.26; p<0.005	right>left	F(1,146)=36.52; p<0.001	WT	female<male	F(1,146)=4.61; p<0.033
						female	APP/PS1>WT	F(1,146)=6.36; p<0.013
**Month 4**	**CTX**			right>left	F(1,145)=7.15; p<0.008			
**Month 8**	**CTX**			right>left	F(1,128)=5.27; p<0.023			

Polarized light imaging – Retardance

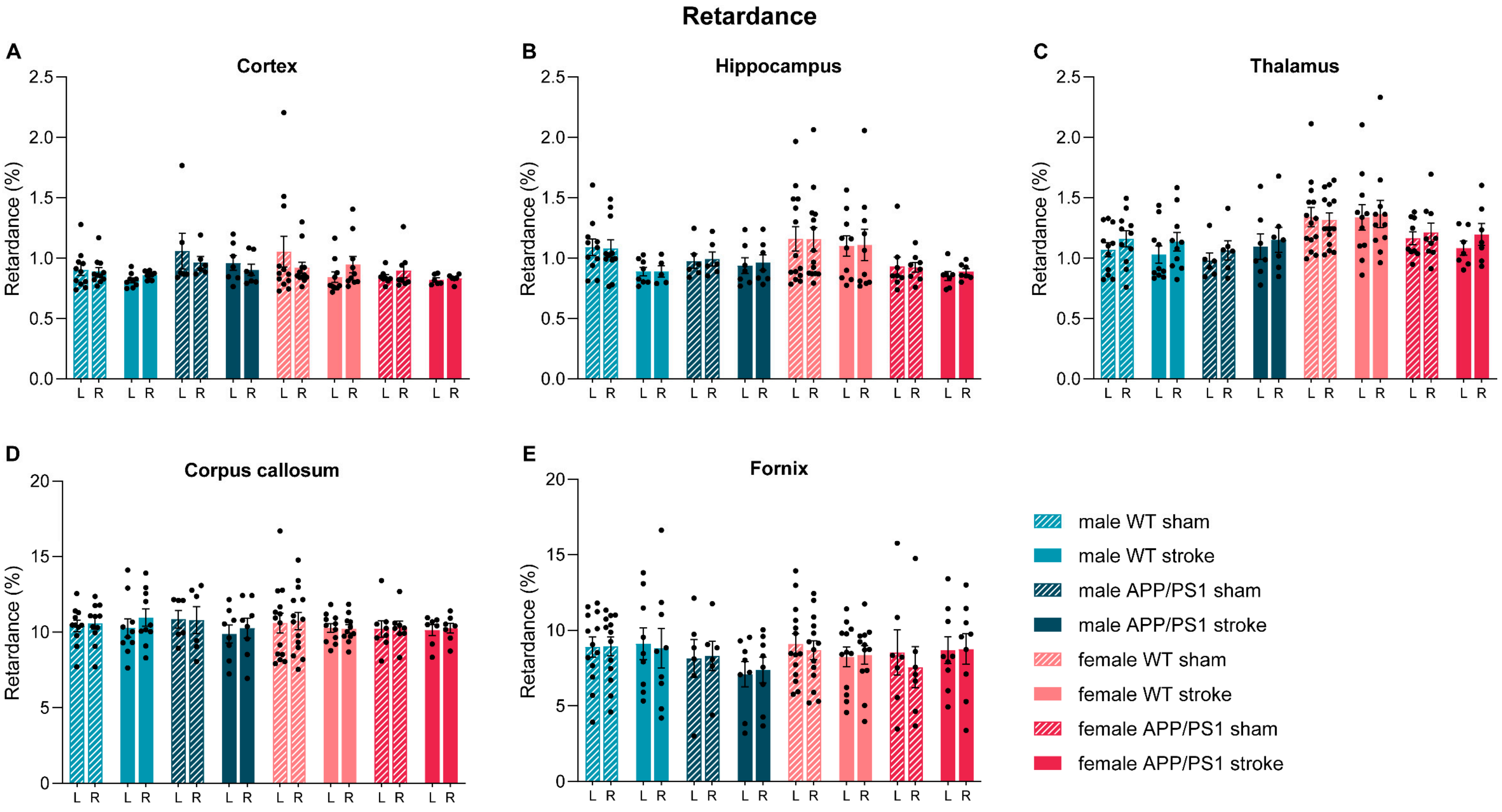

Supplementary Figure S15. Retardance was assessed via polarized light imaging in male and female wild-type (WT) and APP/PS1 mice 8 months post-sham or stroke surgery. Measurements were conducted in the (A) cortex, (B) hippocampus, (C) thalamus, (D) corpus callosum, and (E) fornix. Data are presented as mean ± SEM.

Supplementary Table S31. Raw data of retardance displaying mean ± SEM and group size (n) of male and female wild-type (WT) and APP/PS1 mice 8 months post-stroke or sham surgery. Regions of interest include the cortex (CTX), corpus callosum (CC), fornix (FX), hippocampus (HIP), and thalamus (TH) in the left and right hemispheres.

	male WT sham		male WT stroke		male APP/PS1 sham		male APP/PS1 stroke		female WT sham		female WT stroke		female APP/PS1 sham		female APP/PS1 stroke	
	**mean ± SEM**	**n**	**mean ± SEM**	**n**	**mean ± SEM**	**n**	**mean ± SEM**	**n**	**mean ± SEM**	**n**	**mean ± SEM**	**n**	**mean ± SEM**	**n**	**mean ± SEM**	**n**
**left CTX**	0.903 ± 0.047	11	0.817 ± 0.019	9	1.058 ± 0.148	6	0.959 ± 0.061	7	1.053 ± 0.128	12	0.842 ± 0.044	10	0.843 ± 0.02	8	0.82 ± 0.017	6
**right CTX**	0.889 ± 0.033	11	0.857 ± 0.011	9	0.964 ± 0.049	6	0.903 ± 0.047	7	0.92 ± 0.046	12	0.946 ± 0.068	10	0.897 ± 0.058	8	0.832 ± 0.015	6
**left CC**	10.402 ± 0.384	11	10.267 ± 0.608	10	10.842 ± 0.584	6	9.87 ± 0.592	8	10.589 ± 0.658	14	10.263 ± 0.282	11	10.207 ± 0.54	8	10.127 ± 0.368	7
**right CC**	10.582 ± 0.402	11	10.951 ± 0.571	10	10.788 ± 0.895	6	10.257 ± 0.665	8	10.727 ± 0.575	14	10.208 ± 0.27	11	10.294 ± 0.423	8	10.253 ± 0.329	7
**left FX**	8.89 ± 0.664	13	9.1 ± 1.034	9	8.137 ± 1.245	6	7.096 ± 0.842	8	9.088 ± 0.671	14	8.233 ± 0.653	12	8.529 ± 1.489	7	8.682 ± 0.892	9
**right FX**	8.934 ± 0.617	13	8.818 ± 1.301	9	8.289 ± 0.976	6	7.359 ± 0.842	8	8.668 ± 0.62	14	8.356 ± 0.621	12	7.561 ± 1.357	7	8.749 ± 1.011	9
**left HIP**	1.09 ± 0.068	11	0.888 ± 0.036	8	0.975 ± 0.06	6	0.938 ± 0.065	7	1.16 ± 0.101	14	1.1 ± 0.085	10	0.931 ± 0.077	8	0.851 ± 0.037	7
**right HIP**	1.08 ± 0.072	11	0.889 ± 0.047	5	0.993 ± 0.058	6	0.963 ± 0.065	7	1.156 ± 0.096	14	1.109 ± 0.129	10	0.923 ± 0.04	8	0.888 ± 0.025	7
**left TH**	1.069 ± 0.067	11	1.03 ± 0.078	10	0.98 ± 0.081	6	1.095 ± 0.104	7	1.34 ± 0.059	14	1.337 ± 0.112	11	1.165 ± 0.078	9	1.082 ± 0.091	7
**right TH**	1.16 ± 0.059	11	1.135 ± 0.072	10	1.064 ± 0.063	6	1.151 ± 0.105	7	1.315 ± 0.08	14	1.365 ± 0.106	11	1.213 ± 0.054	9	1.196 ± 0.058	7

Supplementary Table S32. Analysis of retardance using linear mixed model analysis, highlighting all significant effects of fixed factors (sex, genotype, surgery) and their respective interactions. Regions of interest include the cortex (CTX), corpus callosum (CC), fornix (FX), hippocampus (HIP), and thalamus (TH) in the left and right hemispheres. The table specifies the direction of each effect. F-values, degrees of freedom, and p-values are included for each effect.

	Sex		Genotype	
	**Direction**	**p-value**	**Direction**	**p-value**
**left HIP**			APP/PS1<WT	F(1,70)=6.91; p<0.011
**left TH**	female>male	F(1,73)=12.71; p<0.001		
**right TH**	female>male	F(1,73)=6.63; p<0.012		

Supplementary Table S33. Analysis of retardance using linear mixed model analysis, highlighting all significant effects of fixed factors (sex, genotype, surgery, left vs. right hemispheres) and their respective interactions. Regions of interest include the combined cortex (CTX), corpus callosum (CC), fornix (FX), hippocampus (HIP), and thalamus (TH). The table specifies the direction of each effect. F-values, degrees of freedom, and p-values are included for each effect.

	Sex		Genotype		Sex*Genotype		
	**Direction**	**p-value**	**Direction**	**p-value**	**Direction**		**p-value**
**CTX**					APP/PS1	female<male	F(1,133)=5.72; p<0.018
					male	APP/PS1>WT	F(1,133)=4.76; p<0.031
					female	APP/PS1<WT	F(1,133)=4.25; p<0.041
**HIP**			APP/PS1<WT	F(1,135)=10.58; p<0.001			
**TH**	female>male	F(1,147)=19.66; p<0.001	APP/PS1<WT	F(1,147)=6.22; p<0.014			

Polarized light imaging – Dispersion

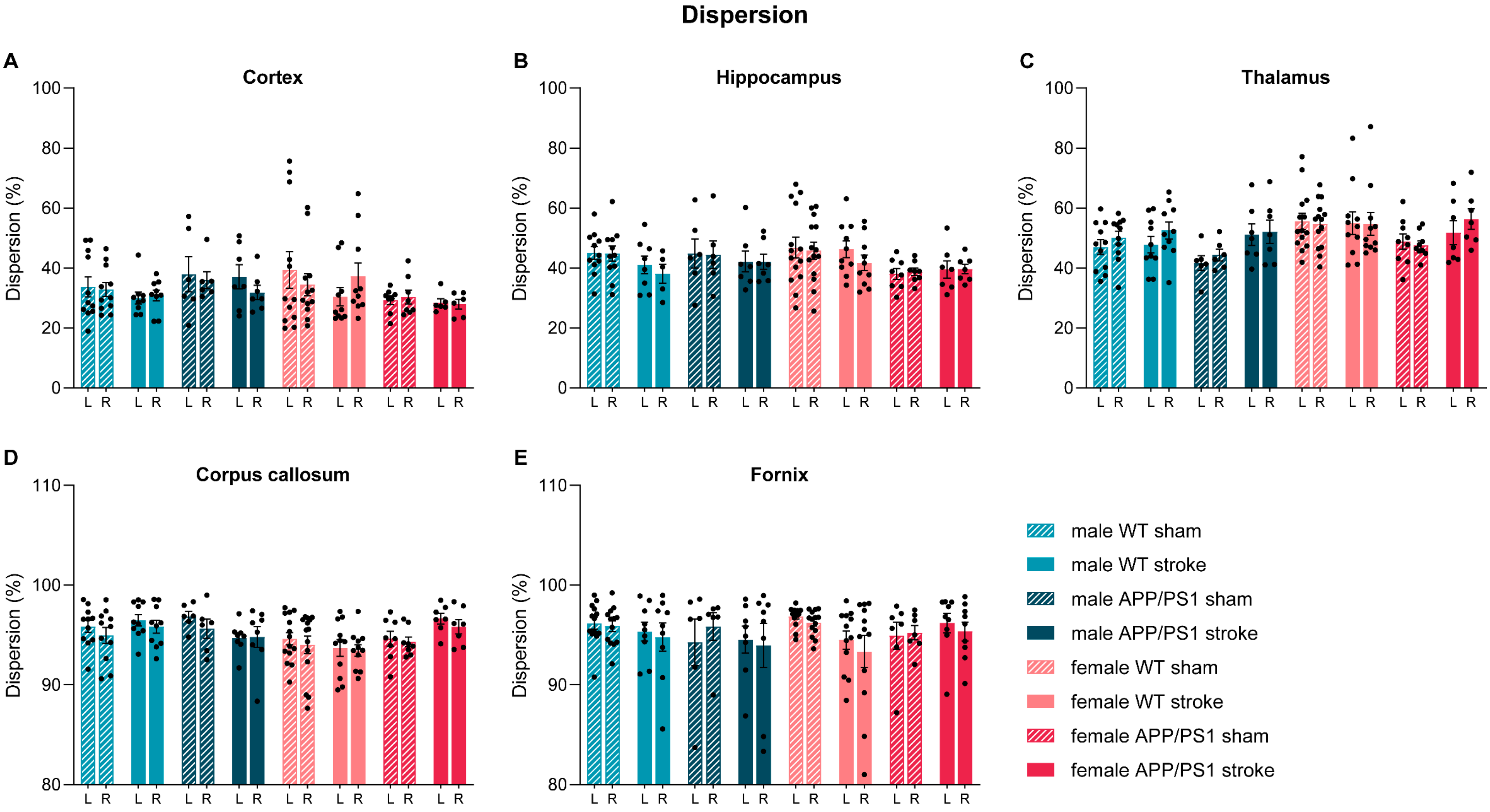

Supplementary Figure S16. Dispersion was assessed via polarized light imaging in male and female wild-type (WT) and APP/PS1 mice 8 months post-sham or stroke surgery. Measurements were conducted in the (A) cortex, (B) hippocampus, (C) thalamus, (D) corpus callosum, and (E) fornix. Data are presented as mean ± SEM.

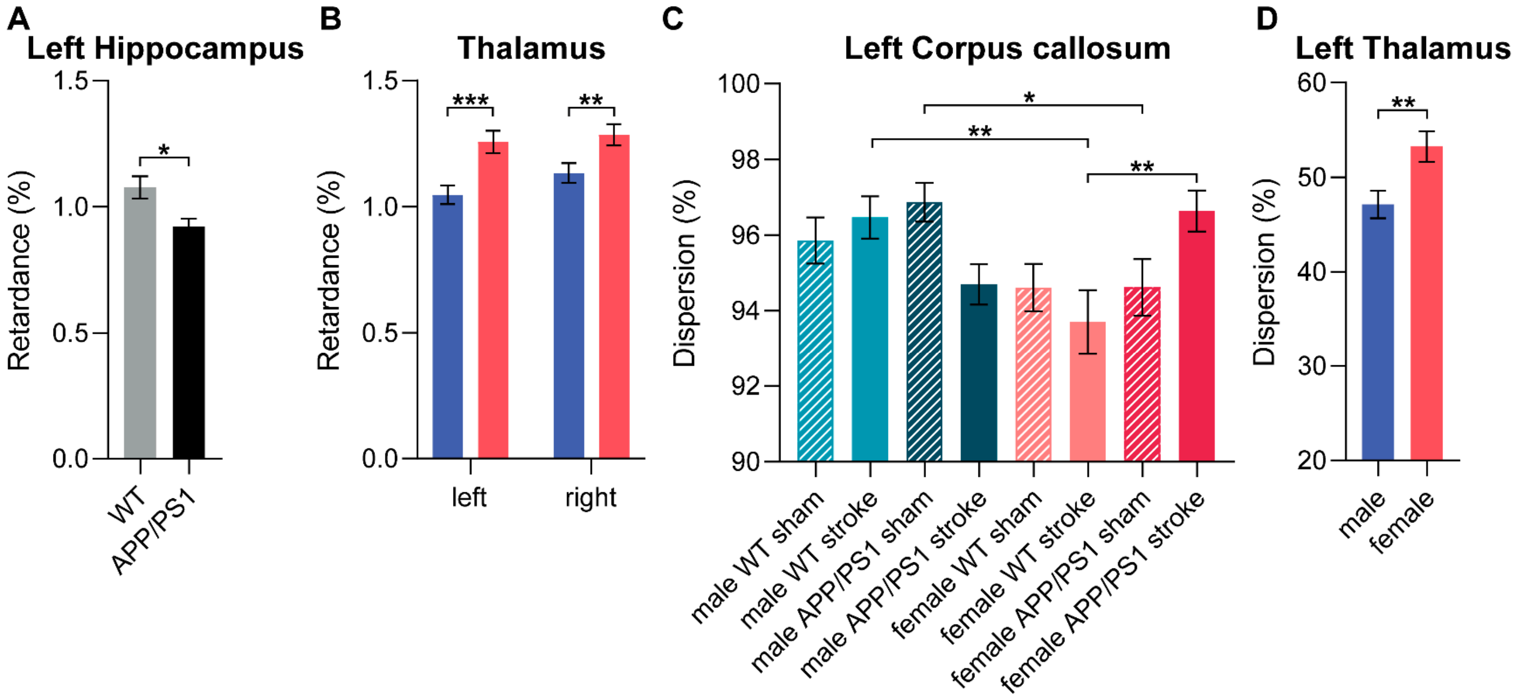

Supplementary Figure S17. Polarized light imaging (PLI) was conducted on post-mortem samples to evaluate myelin density and fiber orientation across different regions of interest, including the cortex, corpus callosum, hippocampus, and thalamus. This analysis utilized retardance maps for myelin density, as shown in the upper panel, and dispersion maps for fiber orientation, as shown in the lower panel. This analysis was carried out 8 months after inducing stroke, at which point the animals, encompassing both male and female, sham, and stroke-operated WT and APP/PS1 mice, were 12 months old. The retardance values obtained from PLI serve as indicators of myelination, with lower values suggesting potential myelin degradation. Similarly, dispersion levels offer a quantitative assessment of fiber orientation, where lower dispersion indicates better myelin quality. (A) In the left hippocampus, APP/PS1 mice displayed lower myelin density compared to WT mice, highlighting a clear genotype effect (WT n=43, APP/PS1 n=28). (B) The thalamus exhibited a sex effect, with female mice consistently showing higher myelin density than male mice in both hemispheres (left male n=34, right male n=34, left female n=41, right female n=41). (C) In the left corpus callosum, a complex interaction involving sex, genotype, and surgery type was observed. WT stroke and APP/PS1 sham male mice had significantly higher dispersion than their female counterparts. Notably, stroke-operated APP/PS1 female mice displayed higher dispersion values than APP/PS1 sham females (male WT sham n=11, male WT stroke n=10, male APP/PS1 sham n=6, male APP/PS1 stroke n=8, female WT sham n=14, female WT stroke n=11, female APP/PS1 sham n=8, female APP/PS1 stroke n=7). (D) Furthermore, in the left thalamus, dispersion levels were generally higher in female mice compared to male mice (male n=34, female n=41). Data are presented as mean ± SEM. Significance is denoted as *$p < 0.05$, **$p < 0.01$, ***$p < 0.001$.

Supplementary Table S34. Raw data of dispersion displaying mean ± SEM and group size (n) of male and female wild-type (WT) and APP/PS1 mice 8 months post-stroke or sham surgery. Regions of interest include the cortex (CTX), corpus callosum (CC), fornix (FX), hippocampus (HIP), and thalamus (TH) in the left and right hemispheres.

	male WT sham		male WT stroke		male APP/PS1 sham		male APP/PS1 stroke		female WT sham		female WT stroke		female APP/PS1 sham		female APP/PS1 stroke	
	mean ± SEM	n	mean ± SEM	n	mean ± SEM	n	mean ± SEM	n	mean ± SEM	n	mean ± SEM	n	mean ± SEM	n	mean ± SEM	n
**left CTX**	33.723 ± 3.331	11	29.991 ± 1.981	9	37.918 ± 5.829	6	37.099 ± 4.012	7	39.305 ± 6.082	12	30.404 ± 3.033	10	29.201 ± 1.459	8	28.416 ± 1.335	6
**right CTX**	32.779 ± 2.321	11	30.833 ± 1.818	9	35.973 ± 2.828	6	31.805 ± 2.374	7	34.387 ± 3.631	12	37.157 ± 4.46	10	30.262 ± 2.293	8	27.928 ± 1.587	6
**left CC**	95.854 ± 0.608	11	96.464 ± 0.558	10	96.868 ± 0.512	6	94.695 ± 0.536	8	94.607 ± 0.627	14	93.694 ± 0.848	11	94.616 ± 0.752	8	96.636 ± 0.541	7
**right CC**	94.951 ± 0.791	11	95.829 ± 0.653	10	95.62 ± 0.951	6	94.791 ± 1.043	8	93.999 ± 0.881	14	93.455 ± 0.576	11	94.295 ± 0.499	8	95.809 ± 0.709	7
**left FX**	96.168 ± 0.589	13	95.33 ± 0.931	9	94.259 ± 2.333	6	94.522 ± 1.342	8	96.843 ± 0.267	14	94.496 ± 0.908	12	94.921 ± 1.376	7	96.184 ± 0.982	9
**right FX**	95.864 ± 0.535	13	94.767 ± 1.421	9	95.819 ± 1.377	6	93.94 ± 2.205	8	96.207 ± 0.337	14	93.335 ± 1.626	12	95.212 ± 0.721	7	95.35 ± 0.944	9
**left HIP**	45.021 ± 2.173	11	41.02 ± 3.007	8	44.831 ± 4.91	6	42.104 ± 3.492	7	46.782 ± 3.511	14	46.237 ± 2.808	10	38.049 ± 1.806	8	39.54 ± 2.866	7
**right HIP**	44.775 ± 2.569	11	38.072 ± 3.197	5	44.337 ± 4.6	6	42.07 ± 2.527	7	45.795 ± 2.829	14	41.679 ± 2.603	10	38.707 ± 1.23	8	39.617 ± 1.584	7
**left TH**	46.928 ± 2.45	11	47.837 ± 2.752	10	41.654 ± 2.437	6	51.076 ± 3.618	7	55.512 ± 2.685	14	54.942 ± 3.746	11	48.76 ± 2.59	9	51.74 ± 4.018	7
**right TH**	50.075 ± 2.201	11	52.524 ± 2.861	10	44.378 ± 1.967	6	52.078 ± 3.871	7	54.676 ± 2.145	14	54.681 ± 3.783	11	47.583 ± 1.412	9	56.3 ± 3.379	7

Supplementary Table S35. Analysis of dispersion using linear mixed model analysis, highlighting all significant effects of fixed factors (sex, genotype, surgery) and their respective interactions. Regions of interest include the cortex (CTX), corpus callosum (CC), fornix (FX), hippocampus (HIP), and thalamus (TH) in the left and right hemispheres. The table specifies the direction of each effect. F-values, degrees of freedom, and p-values are included for each effect.

	Sex		Sex*Genotype*Surgery			
	Direction	p-value	Direction			p-value
left CC			WT	stroke	female<male	F(1,67)=9.37; p<0.003
			APP/PS1	sham	female<male	F(1,67)=4.05; p<0.048
			female	stroke	APP>WT	F(1,67)=8.63; p<0.005
left TH	female>male	F(1,73)=7.59; p<0.007				

Supplementary Table S36. Analysis of dispersion using linear mixed model analysis, highlighting all significant effects of fixed factors (sex, genotype, surgery, left vs. right hemispheres) and their respective interactions. Regions of interest include the combined cortex (CTX), corpus callosum (CC), fornix (FX), hippocampus (HIP), and thalamus (TH). The table specifies the direction of each effect. F-values, degrees of freedom, and p-values are included for each effect.

	Sex		Surgery		Sex*Genotype		
	Direction	p-value	Direction	p-value	Direction		p-value
CTX					APP/PS1	female<male	F(1,134)=5.14; p<0.025
					female	APP/PS1<WT	F(1,134)=6.23; p<0.014
CC	female<male	F(1,142)=6.92; p<0.009					
TH	female>male	F(1,145)=11.54; p<0.001	stroke>sham	F(1,145)=6.74; p<0.01			

Neuroinflammation

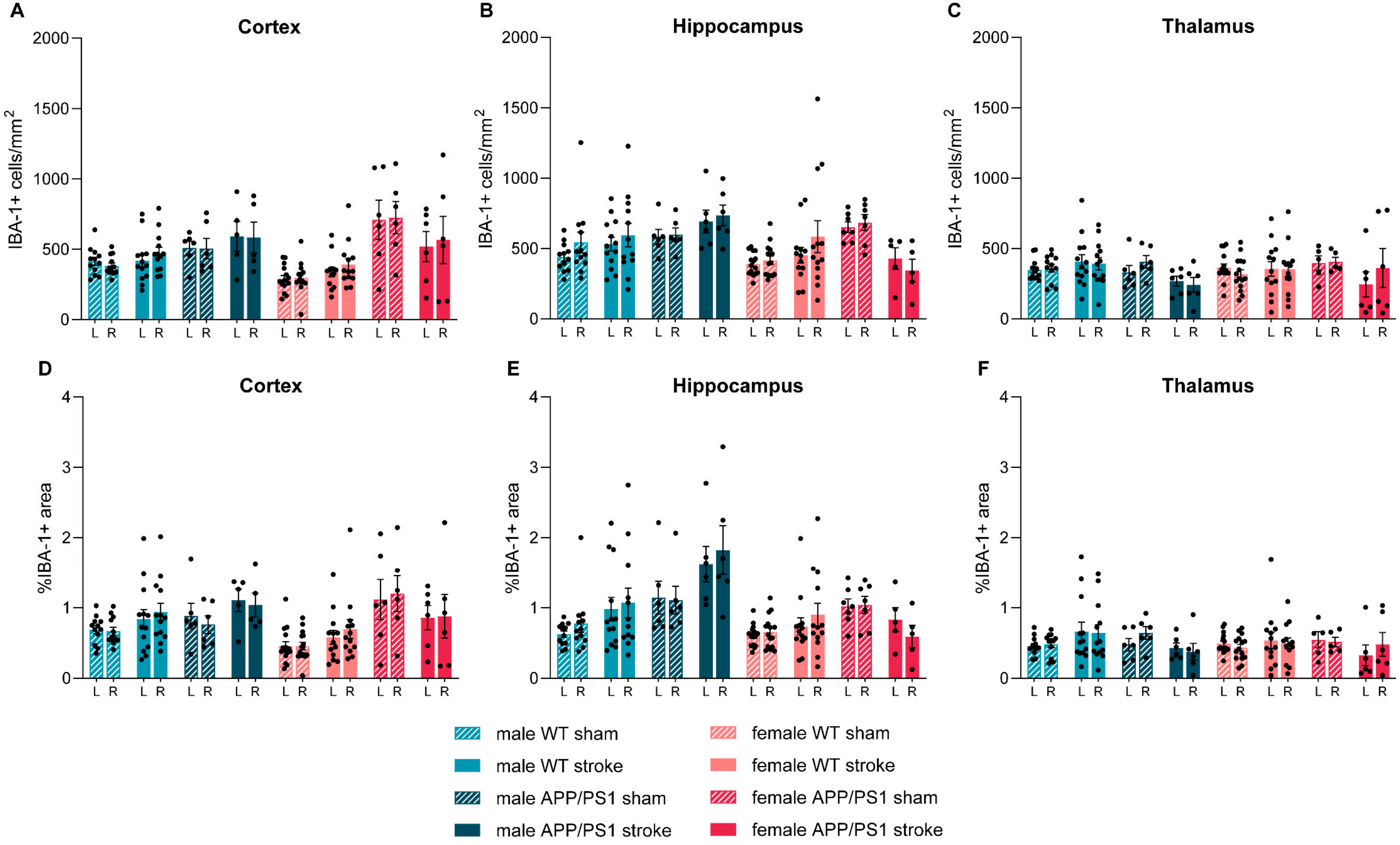

Supplementary Figure S18. Immunohistochemical analysis of ionized calcium-binding adapter molecule 1 (IBA-1) to assess neuroinflammation in male and female wild-type (WT) and APP/PS1 mice, 8 months post-sham or stroke surgery. The figure shows the number of IBA-1+ cells per mm² in the (A) cortex, (B) hippocampus, and (C) thalamus. Additionally, the relative IBA-1+ area is depicted in the (D) cortex, (E) hippocampus, and (F) thalamus. Data are presented as mean ± SEM.

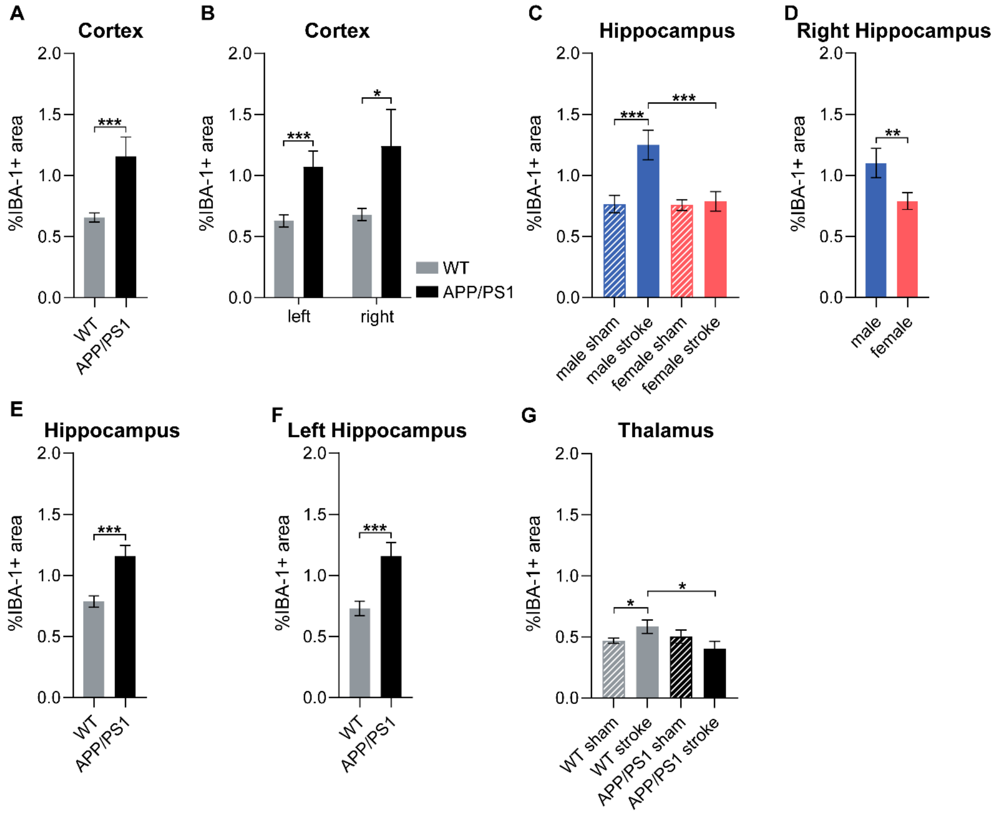

Supplementary Figure S19. Quantification of the Iba-1+ area was performed across the cortex, hippocampus, and thalamus in male and female, sham or stroke-operated WT and APP/PS1 mice, at 12 months of age and 8 months post-surgery. (A) In the cortex, APP/PS1 mice exhibited a larger Iba-1+ area than WT mice (WT n=106, APP/PS1 n=46), (B) a finding detected in both the right and left cortex (right WT n=53, right APP/PS1 n=23, left WT n=53, left APP/PS1 n=23). (C) In the hippocampus, male stroke mice exhibited a larger Iba-1+ area in comparison to male sham and female stroke mice (male sham n=36, male stroke n=37, female sham n=44, female stroke n=36). (D) In the right hippocampus, female mice displayed a smaller Iba-1+ area compared to male mice (male n=36, female n=40). (E) Similarly to the genotype effect in the cortex, in the hippocampus APP/PS1 mice displayed a larger Iba-1+ area than WT mice (WT n=105, APP/PS1 n=48), (F) particularly in the left hippocampus (WT n=53, APP/PS1 n=24). (G) In the thalamus, a larger Iba-1+ area was observed in WT stroke mice compared to WT sham mice. Additionally, stroke-operated APP/PS1 mice had a smaller Iba-1+ area than WT stroke mice (WT sham n=52, WT stroke n=52, APP/PS1 sham n=16, APP/PS1 stroke n=24). Data are presented as mean ± SEM. Significance is denoted as *p < 0.05, **p < 0.01, ***p < 0.001.

Supplementary Table S37. Raw data of IBA-1+ cells per mm² and relative IBA-1+ area displaying mean ± SEM and group size (n) of male and female wild-type (WT) and APP/PS1 mice 8 months post-stroke or sham surgery. Regions of interest include the cortex (CTX), hippocampus (HIP), and thalamus (TH) in the left and right hemispheres.

		male WT sham		male WT stroke		male APP/PS1 sham		male APP/PS1 stroke		female WT sham		female WT stroke		female APP/PS1 sham		female APP/PS1 stroke	
		**mean ± SEM**	**n**	**mean ± SEM**	**n**	**mean ± SEM**	**n**	**mean ± SEM**	**n**	**mean ± SEM**	**n**	**mean ± SEM**	**n**	**mean ± SEM**	**n**	**mean ± SEM**	**n**
**IBA-1+ cell/mm2**	**left CTX**	414.695 ± 28.634	12	509.245 ± 44.234	13	509.245 ± 44.234	13	592.363 ± 106.505	5	290.263 ± 23.576	15	338.523 ± 33.086	13	709.028 ± 139.898	6	520.197 ± 107.183	6
	**right CTX**	382.239 ± 20.474	12	505.466 ± 39.172	13	505.466 ± 39.172	13	583.133 ± 110.849	5	297.734 ± 27.762	15	391.141 ± 44.144	13	724.777 ± 113.832	6	566.82 ± 167.559	6
	**left HIP**	439.274 ± 29.308	12	584.056 ± 49.036	13	584.056 ± 49.036	13	692.109 ± 81.161	6	390.156 ± 20.665	15	452.474 ± 54.536	13	651.149 ± 38.206	7	429.053 ± 77.593	5
	**right HIP**	542.259 ± 73.717	12	598.839 ± 83.532	12	598.839 ± 83.532	12	734.768 ± 75.312	6	416.279 ± 29.684	15	584.296 ± 113.431	13	684.044 ± 56.151	7	344.302 ± 79.71	5
	**left TH**	348.395 ± 21.578	12	329.721 ± 50.569	13	329.721 ± 50.569	13	269.027 ± 35.615	6	363.83 ± 26.307	15	354.956 ± 52.835	13	395.302 ± 52.792	5	244.948 ± 89.799	6
	**right TH**	361.313 ± 27.607	12	405.789 ± 43.539	13	405.789 ± 43.539	13	242.153 ± 52.232	6	318.623 ± 30.423	15	353.677 ± 49.758	13	405.945 ± 32.624	5	361.286 ± 136.98	6
**%IBA-1+ area**	**left CTX**	0.689 ± 0.057	12	0.884 ± 0.138	13	0.884 ± 0.138	13	1.11 ± 0.16	5	0.455 ± 0.065	15	0.582 ± 0.098	13	1.117 ± 0.282	6	0.859 ± 0.174	6
	**right CTX**	0.668 ± 0.056	12	0.763 ± 0.126	13	0.763 ± 0.126	13	1.04 ± 0.168	5	0.458 ± 0.054	15	0.695 ± 0.132	13	1.202 ± 0.252	6	0.879 ± 0.31	6
	**left HIP**	0.624 ± 0.045	12	1.143 ± 0.167	13	1.143 ± 0.167	13	1.623 ± 0.255	6	0.606 ± 0.04	15	0.736 ± 0.122	13	1.02 ± 0.102	7	0.831 ± 0.169	5
	**right HIP**	0.776 ± 0.122	12	1.106 ± 0.21	12	1.106 ± 0.21	12	1.822 ± 0.347	6	0.656 ± 0.06	15	0.898 ± 0.164	13	1.04 ± 0.12	7	0.591 ± 0.157	5
	**left TH**	0.457 ± 0.038	12	0.478 ± 0.131	13	0.478 ± 0.131	13	0.429 ± 0.068	6	0.493 ± 0.037	15	0.536 ± 0.116	13	0.547 ± 0.113	5	0.326 ± 0.144	6
	**right TH**	0.484 ± 0.046	12	0.643 ± 0.117	13	0.643 ± 0.117	13	0.378 ± 0.118	6	0.433 ± 0.046	15	0.495 ± 0.077	13	0.516 ± 0.067	5	0.479 ± 0.168	6

Supplementary Table S38. Analysis of neuroinflammation (IBA-1+ cells per mm², relative IBA-1+ area) using linear mixed model analysis, highlighting all significant effects of fixed factors (sex, genotype, surgery) and their interactions. Regions of interest include the cortex (CTX), hippocampus (HIP), and thalamus (TH) in the left and right hemispheres. The table specifies the direction of each effect, with F-values, degrees of freedom, and p-values included for each.

		Sex		Genotype		Sex*Genotype			Genotype*Surgery			Sex*Surgery		
		Direction	p-value	Direction	p-value	Direction		p-value	Direction		p-value	Direction		p-value
IBA-1+ cells/mm2	CTX					WT	female<male	F(1,144)=7.52; p<0.007						
						male	APP/PS1>WT	F(1,144)=7.8; p<0.006						
						female	APP/PS1<WT	F(1,144)=49.95; p<0.001						
	HIP	female<male	F(1,145)=7.1; p<0.009	APP/PS1>WT	F(1,145)=7.19; p<0.008									
	TH								stroke	APP/PS1<WT	F(1,148)=7; p<0.009			
									APP/PS1	stroke<sham	F(1,148)=5.53; p<0.02			
%IBA-1+ area	CTX			APP/PS1>WT	F(1,148)=18.61; p<0.001									
	HIP			APP/PS1>WT	F(1,145)=19.05; p<0.001							stroke	female<male	F(1,145)=26.3; p<0.001
												male	stroke>sham	F(1,145)=15.84; p<0.001
	TH								stroke	APP/PS1<WT	F(1,148)=6.49; p<0.012			
									WT	stroke>sham	F(1,148)=4.43; p<0.037			

Supplementary Table S39. Analysis of neuroinflammation (IBA-1+ cells per mm², relative IBA-1+ area) using linear mixed model analysis, highlighting all significant effects of fixed factors (sex, genotype, surgery, left vs. right hemispheres) and their interactions. Regions of interest include the cortex (CTX), hippocampus (HIP), and thalamus (TH). The table specifies the direction of each effect, with F-values, degrees of freedom, and p-values included for each effect.

		**Sex**		**Genotype**		**Sex*Surgery**		
		**Direction**	**p-value**	**Direction**	**p-value**	**Direction**		**p-value**
**IBA-1+ cell/mm2**	**left CTX**			APP/PS1>WT	F(1,74)=25.84; p<0.001			
	**right CTX**			APP/PS1>WT	F(1,74)=19.29; p<0.001			
	**left HIP**			APP/PS1>WT	F(1,69)=13.79; p<0.001	stroke	female<male	F(1,69)=10.33; p<0.002
**%IBA-1+ area**	**left CTX**			APP/PS1>WT	F(1,74)=12.45; p<0.001			
	**right CTX**			APP/PS1>WT	F(1,73)=6.77; p<0.011			
	**left HIP**			APP/PS1>WT	F(1,72)=17.28; p<0.001			
	**right HIP**	female<male	F(1,68)=9.32; p<0.003					

Amyloid beta staining

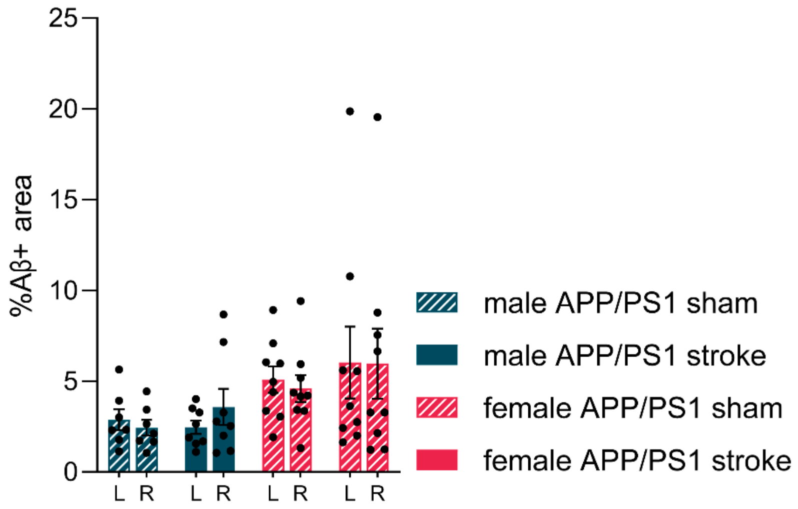

Supplementary Figure S20. Immunohistochemical analysis amyloid beta (Aβ) in male and female APP/PS1 mice, 8 months post-sham or stroke surgery. The figure shows the relative IBA-1+ area in the hippocampus. Data are presented as mean ± SEM.

Supplementary Table S40. Raw data of relative Aβ+ area in the hippocampus displaying mean ± SEM and group size (n) of male and female APP/PS1 mice 8 months post-stroke or sham surgery.

	male APP/PS1 sham		male APP/PS1 stroke		female APP/PS1 sham		female APP/PS1 stroke	
	mean ± SEM	n	mean ± SEM	n	mean ± SEM	n	mean ± SEM	n
**% Aβ + area: left HIP**	2.874 ± 0.57	7	2.463 ± 0.372	8	5.08 ± 0.727	9	6.023 ± 1.97	9
**% Aβ + area: right HIP**	2.433 ± 0.44	7	3.579 ± 0.993	8	4.593 ± 0.741	9	5.964 ± 1.933	9

Supplementary Table S41. Analysis of amyloid beta staining of the hippocampus (% Aβ+ area) using linear mixed model analysis, highlighting all significant effects of fixed factors (sex, genotype, surgery, left vs. right hemispheres) and their interactions. The table specifies the direction of each effect, with F-values, degrees of freedom, and p-values included for each effect.

	Sex	
	Direction	p-value
**% Aβ+ area**	female>male	$F(1,64)=9.43$; $p<0.003$

Aβ ELISA

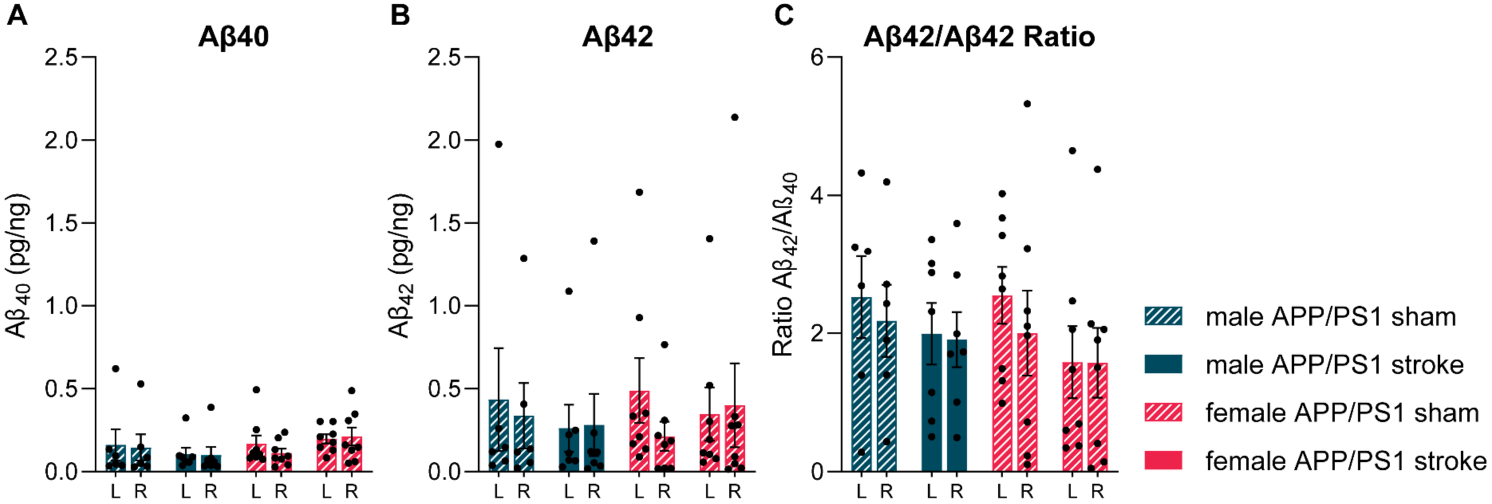

Supplementary Figure S21. Brain tissue levels of (A) Aβ40, (B) Aβ42, and (C) Aβ42/Aβ40 ratio in male and female APP/PS1 mice, 8 months post-sham or stroke surgery in the left and right hemispheres. Data are presented as mean ± SEM.

Supplementary Table S42. Raw data of $A\beta_{40}$, $A\beta_{42}$, and $A\beta_{42}/A\beta_{40}$ ratio measurements displaying mean ± SEM and group size (n) of male and female APP/PS1 mice 8 months post-stroke or sham surgery. Regions of interest include the cortex (CTX), hippocampus (HIP), and thalamus (TH) in the left and right hemispheres.

	male APP/PS1 sham		male APP/PS1 stroke		female APP/PS1 sham		female APP/PS1 stroke	
	mean ± SEM	n	mean ± SEM	n	mean ± SEM	n	mean ± SEM	n
left $A\beta_{40}$	0.161 ± 0.093	6	0.107 ± 0.037	7	0.168 ± 0.05	8	0.198 ± 0.028	8
right $A\beta_{40}$	0.146 ± 0.079	6	0.101 ± 0.048	7	0.112 ± 0.026	8	0.212 ± 0.054	8
left $A\beta_{42}$	0.434 ± 0.31	6	0.262 ± 0.141	7	0.488 ± 0.195	8	0.347 ± 0.161	8
right $A\beta_{42}$	0.337 ± 0.197	6	0.282 ± 0.187	7	0.213 ± 0.087	8	0.4 ± 0.252	8
left ratio $A\beta_{42}/A\beta_{40}$	2.521 ± 0.592	6	1.991 ± 0.445	7	2.547 ± 0.409	8	1.582 ± 0.52	8
right ratio $A\beta_{42}/A\beta_{40}$	2.177 ± 0.521	6	1.908 ± 0.397	7	2 ± 0.613	8	1.572 ± 0.505	8
